# Radiotherapy-sensitized cancer immunotherapy via cGAS-STING immune pathway by activatable nanocascade reaction

**DOI:** 10.1186/s12951-024-02502-8

**Published:** 2024-05-09

**Authors:** Honglei Hu, Shuting Zheng, Chenxi He, Yinfei Zheng, Qiming Wei, Siwen Chen, Zede Wu, Yikai Xu, Bingxia Zhao, Chenggong Yan

**Affiliations:** 1 Department of Medical Imaging Center, Nanfang Hospital, Southern Medical University, Guangzhou, 510515 China; 2 Guangzhou Key Laboratory of Tumor Immunology Research, Cancer Research Institute, School of Basic Medical Sciences, Southern Medical University, Guangzhou, 510515 China; 3 Department of Radiology, The Second Affiliated Hospital of Guangzhou Medical University, Guangzhou, 510260 China; 4 Department of Invasive Interventional, The Second Affiliated Hospital of Guangzhou University of Chinese Medicine, Guangzhou, 510120 China

**Keywords:** cGAS-STING, Ferroptosis, Radiotherapy sensitization, Theranostics, Molecular imaging

## Abstract

Radiotherapy-induced immune activation holds great promise for optimizing cancer treatment efficacy. Here, we describe a clinically used radiosensitizer hafnium oxide (HfO_2_) that was core coated with a MnO_2_ shell followed by a glucose oxidase (GOx) doping nanoplatform (HfO_2_@MnO_2_@GOx, HMG) to trigger ferroptosis adjuvant effects by glutathione depletion and reactive oxygen species production. This ferroptosis cascade potentiation further sensitized radiotherapy by enhancing DNA damage in 4T1 breast cancer tumor cells. The combination of HMG nanoparticles and radiotherapy effectively activated the damaged DNA and Mn^2+^-mediated cGAS-STING immune pathway in vitro and in vivo. This process had significant inhibitory effects on cancer progression and initiating an anticancer systemic immune response to prevent distant tumor recurrence and achieve long-lasting tumor suppression of both primary and distant tumors. Furthermore, the as-prepared HMG nanoparticles “turned on” spectral computed tomography (CT)/magnetic resonance dual-modality imaging signals, and demonstrated favorable contrast enhancement capabilities activated by under the GSH tumor microenvironment. This result highlighted the potential of nanoparticles as a theranostic nanoplatform for achieving molecular imaging guided tumor radiotherapy sensitization induced by synergistic immunotherapy.

**Supplementary Information:**

The online version contains supplementary material available at 10.1186/s12951-024-02502-8.

## Introduction

Breast cancer is one of the most common malignant tumors in women, with its incidence rate increasing annually [1, 2]. Despite remarkable advancements in modern medicine, the prognosis for breast cancer patients remains unfavorable [3, 4]. Therefore, it is essential to develop new therapeutic strategies to improve the survival rate for cancer patients. Immunotherapy has emerged as a prominent area of research in tumor treatment in recent years, aiming to stimulate the immune system to combat tumor cells [5–7]. Immunotherapy offers distinct advantages by overcoming immune escape and drug resistance, challenges while prioritizing safety. However, the application of immunotherapy in breast cancer treatment still encounters difficulties, especially in identifying suitable treatment strategies.

An important intracellular cGAS-STING immune pathway has recently been discovered [8–11]. In tumor cells, the released DNA in the cytoplasm can be sensed by cGAS, which triggers the activation of the cGAS-STING pathway activation and subsequently induces an immune response [12–14]. Studies [15–17] have shown that the cGAS-STING pathway can sense manganese ions (Mn^2+^) in the cytoplasm and promote antigen presentation of the tumor cells, improving the recognition and attack capabilities of the immune cells. Consequently, increasing the levels of DNA and Mn^2+^ in tumor cells to activate the cGAS-STING immune pathway holds significant potential for cancer immunotherapy.

Radiotherapy (RT) is a critical therapeutic modality in tumor treatment, which can directly or indirectly induce DNA damage in tumor cells [18, 19]. Several studies [20–23] have discovered that RT not only controls local primary tumors but also regulates the immune response, thereby preventing tumor recurrence and metastasis. However, problems such as radiation tolerance significantly impede the efficacy of RT alone in killing tumor cells [24]. Encouragingly, a recent correlation has been identified between ferroptosis and RT resistance [25–27]. Ferroptosis is a novel form of cell death associated with the metabolism of iron ions and reactive oxygen species (ROS). Previous studies [28–31] have shown that glutathione (GSH) depletion and ROS generation can promote ferroptosis, with a sensitizing effect on RT. This results in increased DNA damage in tumor cells and activates the cGAS-STING immune pathway. Therefore, ferroptosis sensitization by RT shows significant promise for breast cancer immunotherapy.

**Scheme 1:**
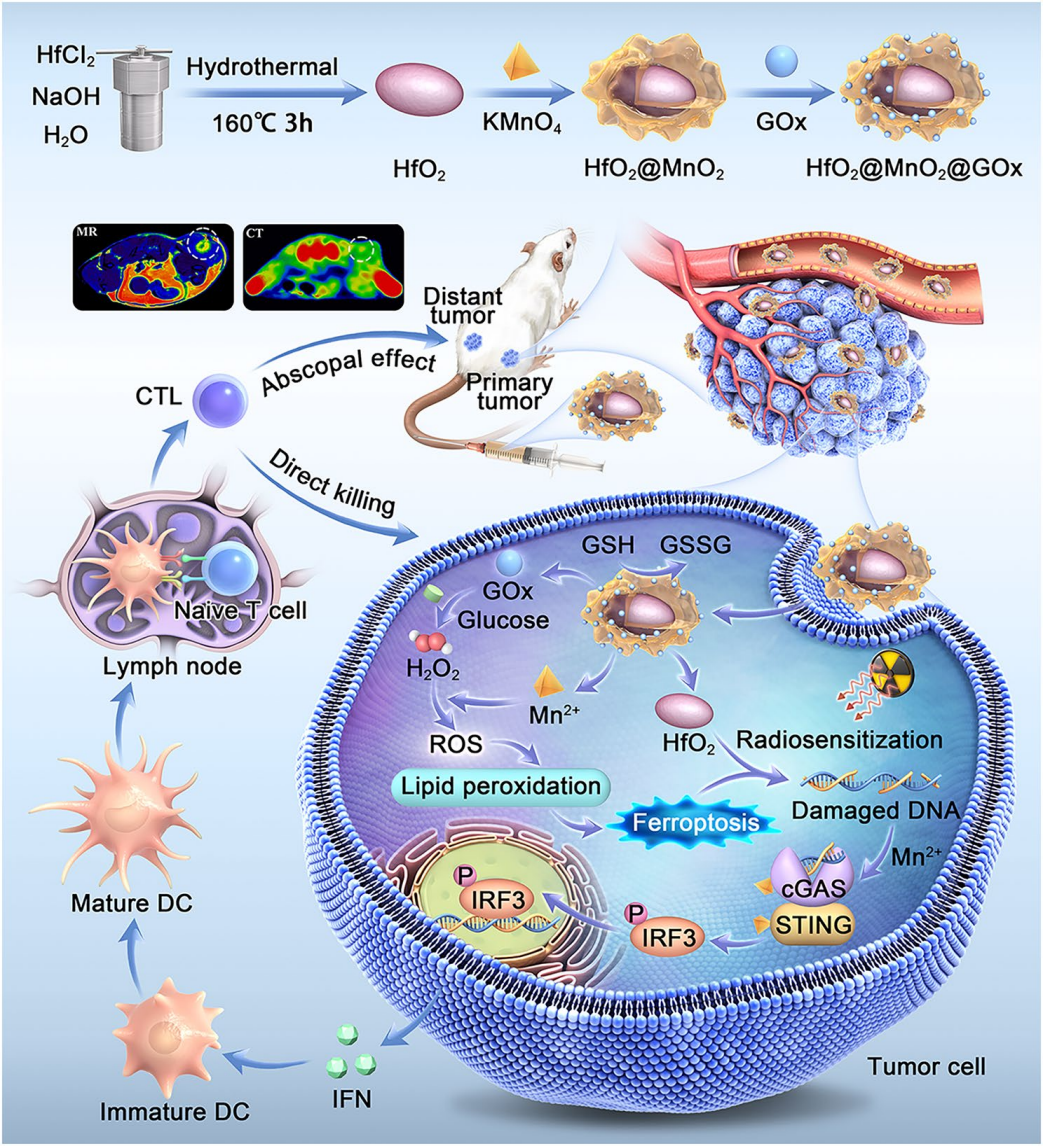
Schematic illustration of the mechanism of HfO_2_@MnO_2_@GOx nanoparticles in the combination therapy of breast cancer

Hafnium oxide (HfO_2_) nanoparticles have been utilized as tumor RT sensitizers in clinical practice [32, 33]. However their sensitization efficiency can be improved [34, 35]. To achieve this optimization in the present study, manganese oxide (MnO_2_) and glucose oxidase (GOx) were sequentially modified on the HfO_2_ core to form HfO_2_@MnO_2_@GOx (HMG) nanoparticles (Scheme 1). The MnO_2_ shell layer was designed to deplete GSH through an oxidation reaction and increase intracellular ROS content via the Fenton-like reaction involving released Mn^2+^. Moreover, GOx catalyzes glucose in tumor cells to generate hydrogen peroxide (H_2_O_2_), which may further facilitate ROS production [36, 37]. The combined capacities of GSH depletion and ROS increase in HMG nanoparticles were studied to demonstrate the potential of ferroptosis induction in order to sensitize RT to boost DNA damage in breast cancer cells. Subsequently, the released DNA and Mn^2+^ were expected to jointly activate the cGAS-STING immune pathway, resulting in combination radioimmunotherapy for breast cancer. In addition, contrast enhanced capabilities of the HMG nanoparticles were investigated for spectral computed tomography (CT) and magnetic resonance imaging (MRI) in vitro and in vivo.

## Materials and methods

### Materials

Hafnium chloride (HfCl_4_, 99%), polyacrylic acid (PAA), 3-aminopropyltrimethoxysilane (APTES), 1-(3-dimethylaminopropyl)-3-ethylcarbodiimide hydrochloride (EDC), N-hydroxysuccinimide (NHS), Ferrostatin-1 (Fer-1), and buthionine sulfoximine (BSO) were purchased from Shanghai Macklin Biochemical Technology Co., Ltd. GOx was purchased from Shanghai Aladdin Biochemical Technology Co., Ltd. All other chemicals were of analytical grade or higher purity.

### HfO_2_ (H) nanoparticle synthesis

To prepare the HfCl_4_ solution, 0.24 g of HfCl_4_ was dissolved in 15 mL of deionized water. The reaction was conducted at 80℃ for 60 min. Then, a 0.5% NaOH solution (15 mL) was gradually added to the system at room temperature and stirred for 30 min. Subsequently, all of the mixed solutions were transferred to a stainless steel high-pressure reaction flask and heated at 160℃ for 3 h. After the reaction was finished, it was allowed to cool to room temperature and then washed multiple times with aqueous ethanol and deionized water by centrifugation, before finally being collected in deionized water.

### HfO_2_@MnO_2_ (HM) nanoparticle synthesis

Initially, polyacrylic acid (PAA) was utilized to modify the surface charge of HfO_2_ nanoparticles. A 10 mg/mL HfO_2_ aqueous solution (10 mg) was added dropwise to a 10 mg/mL PAA aqueous solution (10 mL). The mixture was stirred at room temperature for 30 min at a constant speed. The resulting solution was then subjected to centrifugal washing with deionized water. Next, a solution of potassium permanganate (KMnO_4_: 50 mg) was prepared using deionized water. Subsequently, the HfO_2_-PAA solution was slowly added to the potassium permanganate water solution, and the reaction was carried out at room temperature for 30 min. The resulting solution was further subjected to centrifugal washing with deionized water multiple times. The obtained product was then dissolved in anhydrous ethanol and stored for future use.

### HfO_2_@MnO_2_@GOx (HMG) nanoparticle synthesis

Firstly, the HM nanoparticles were modified with amino groups. The HM dissolved in absolute ethanol was placed in a flask and stirred at room temperature. Then, a solution containing 0.4 mL of ammonia water and 60 µL of APTES was added dropwise, followed by a reaction at room temperature for 4 h with constant stirring. The resulting solution was then centrifuged, and washed with deionized water.

Next, the carboxyl group of GOx was activated under light-free conditions. A solution containing 10 mg of GOx, 32 mg of NHS, and 40 mg of EDC was prepared in 10 mL of deionized water and reacted at room temperature with stirring at 500 rpm for 30 min in the absence of light.

Finally, the amino group-modified HM aqueous solution was slowly added dropwise to the GOx solution after carboxyl activation. The mixture was continuously stirred at room temperature for 12 h to conduct an amidation reaction to load GOx onto HM. The final product was dissolved in deionized water and stored at 4 °C for subsequent experiments.

### Characterization

The morphology and elemental composition of the nanoparticles were analyzed using a transmission electron microscope (TEM) and a high-resolution TEM (JEM 2100 F, Japan). The surface charge of the nanoparticles was determined using a Zeta PALS system (Brookhaven Instruments, Holtsville, NY, USA). The absorption spectra of the nanoparticles were measured using an ultraviolet-visible spectrophotometer (Agilent Cary 60, USA). The elemental concentration of the nanoparticles was determined using inductively-coupled plasma optical emission spectroscopy (ICP-OES). The imaging performance of the nanoparticles was evaluated using a spectral CT scanner (GE Revolution, USA) and a 3.0T magnetic resonance scanner (Philips Ingenia, the Netherlands).

### ROS detection

The absorbance of the final solution at 665 nm was measured using an ultraviolet-visible spectrophotometer to determine the ROS concentration in the reaction system. After adding Methylene blue (MB) (10 µg/mL), nanoparticles (15 µg/mL, Mn), and H_2_O_2_ (10 µM), various concentrations of GSH (0, 0.1, 0.5, and 1 mM) were used. To investigate the relationship between ROS production in the reaction system and the concentration of H_2_O_2_, the groups were designated as nano + MB + GSH, nano + MB + GSH + H_2_O_2_ (5 µM), and nano + MB + GSH + H_2_O_2_ (10 µM). The MB concentration in the reaction system was 10 µg/mL, while the GSH concentration was 1 mM.

### GOx activity determination

The groups used to evaluate gluconic acid (Glu) included Glu as the positive control, HM + Glu, and HMG + Glu. Nanoparticle concentration was defined based on the Mn element content (15 µg/mL). Solution I consisted of 14.6 mg of ethylenediamine tetraacetic acid and 0.75 µL of trimethylamine (2 M) in 10 mL of deionized water. Solution II involved mixing 178 µL of hydroxylamine with 822 µL of deionized water. Solution III was prepared by adding 162 mg of iron (III) chloride (FeCl_3_), 408.5 mg of trichloroacetic acid, and 833 µL of hydrochloric acid (HCl) to 9.167 mL of deionized water. After 12-h reaction, each sample was centrifuged to obtain 1.6 mL of supernatant. Then, 500 µL of solution I and 50 µL of solution II were added separately to the supernatant and mixed at 37℃ for 25 min. Afterwards, 250 µL of solution III was added to the mixture, and the spectral absorption was immediately measured using an ultraviolet spectrophotometer. Indirectly, the comparison of spectral absorption can confirm whether GOx with enzyme activity was successfully loaded onto the nanoparticles and whether the synthesis of HMG nanoparticles was successful.

The detection of H_2_O_2_ was performed using a Solarbio H_2_O_2_ content detection kit. The experimental groups included Glu water solution, Glu + HM, and Glu + HMG, with different reaction time points (0, 15, 30, 60, 120, 180, and 240 min).

### Release of Mn^2+^ from HMG nanoparticles

The same concentration of HMG (Mn: 15 µg/mL) was used in the reaction system with different pH, H_2_O_2_, and GSH conditions (pH: 6.5/7.4, H_2_O_2_: 10 mM, GSH: 1 mM). Then, 1 mL of the reaction solution was taken at different time points (0, 5, 10, 20, 30, and 60 min) and centrifuged to remove the supernatant. The sample’s Mn^2+^ content was measured using ICP-OES.

### Evaluation of the oxygen production from HMG nanoparticles

The same concentration of HMG (Mn: 15 µg/mL) was used in the reaction system with different pH, H_2_O_2_, and GSH conditions (pH: 6.5/7.4, H_2_O_2_: 10 mM, GSH: 1 mM). Then at pre-determined time points, the oxygen concentration was measured with the dissolved oxygen meter (DO200, Clean, China).

### Cytotoxicity experiment

The cell lines used in the study included 3T3 mouse embryonic fibroblasts and 4T1 mouse breast cancer cells and were obtained from the American Type Culture Collection. These cells were cultured in Dulbecco’s Modified Eagle medium supplemented with 10% fetal bovine serum.

The cells were seeded onto 96-well plates and incubated at 37℃ with 5% CO_2_ for 24 h. Subsequently, the medium was replaced with fresh medium containing nanoparticles and the cells were further cultured for another 24 h. Finally, the cell viability was assessed by the CCK-8 kit to measure the absorbance at 450 nm.

### Intracellular GSH detection

The study sample were divided into four groups: PBS, H, HM, and HMG. Each group consisted of 4T1 cells seeded at a density of 1.5 × 10^4^ cells per well within six-well plates. The cells were cultured for 24 h in a CO_2_ incubator. Subsequently, the pre-existing medium was removed and fresh medium containing various nanoparticles (PBS, H, HM, and HMG) was added to each well for another 6 h. The Hf and Mn concentrations were set at 1 µg/mL and 5 µg/mL, respectively. After incubation, 1 × 10^4^ cells were collected from each group for GSH content analysis following the Solarbio GSH content detection kit instructions.

### Intracellular ROS analysis

The 4T1 cells were divided into four groups: PBS, H, HM, and HMG. These cells were seeded in 96-well plates at a density of 8,000 cells per well and incubated at 37℃ with 5% CO_2_ in a cell culture incubator for 24 h. After that, the pre-existing medium was replaced with fresh medium containing either PBS or nanoparticles (Hf: 1 µg/mL, Mn: 5 µg/mL) and cultured for another 6 h. Next, ROS fluorescence detection reagent was used to stain the intracellular ROS. Finally, ROS content was evaluated using an inverted fluorescence microscope and flow cytometry.

### Evaluation of cellular lipid peroxidation

A BODIPY 581/591 C11 probe was used to analyze lipid peroxidation. The specific procedure involved seeding 4T1 cells at a density of 8,000 or 1.5 × 10^4^ cells per well in 96-well or six-well plates and culture for 24 h. Next, the medium was replaced with fresh medium containing different nanomaterials (Hf: 1 µg/mL, Mn: 5 µg/mL) for 24 h. Then, the cells were cultured in a basic medium containing 1 µM BODIPY 581/591 C11 fluorescent dye for 30 min in the dark, and finally evaluated using an inverted fluorescence microscope and flow cytometry.

### Cell feedback experiment

Ferroptosis inhibitor Ferrostatin-1 (Fer-1) was used to investigate whether 4T1 cell cytotoxicity induced by nanoparticles was mediated by ferroptosis. First, 8,000 4T1 cells were seeded in 96-well plates and incubated for 24 h. Then, the used medium was replaced with medium containing either 10 µM Fer-1 or 100 µM BSO, and incubated for 6 h. Subsequently, the cells were treated with a medium containing nanoparticles and cultured for 24 h. Finally, CCK-8 reagent was added to the wells, and an enzyme-labeled instrument was used to assess cell viability.

### Western blot assay

Protein lysate was prepared from tumor tissues or cells for protein extraction. The proteins were transferred to polyvinylidene difluoride membranes and immunoblotting was performed using antibodies. Protein levels were then analyzed using the GeneSnap system.

### Colony formation

The 4T1 cells were seeded in a six-well plate at a density of 10,000 cells per well. The medium was replaced after 24 h with medium containing a specific concentration of nanomaterials (Hf: 1 µg/mL, Mn: 5 µg/mL). Incubation was continued for another 24 h. The cells were then exposed to 0, 2, 4, 6, and 8 Gy of X-ray radiation. Next, the cells were incubated at 37 °C for eight days, with medium changed periodically. Finally, the cells were fixed by adding 1 mL of methanol per well for 30 min and stained with 0.5% crystal violet solution for 2 h to observe cell colony formation.

### Cell DNA damage detection in vitro

The experiment consisted of eight groups: PBS, H, HM, HMG, RT, H + RT, HM + RT, and HMG + RT. The concentrations of Mn and Hf were 5 µg/mL and 1 µg/mL, respectively. Special confocal plates were used for cell spreading, with an inoculation density of 1 × 10^4^ 4T1 cells per plate. The cells were incubated for 6 h after the addition of medium containing nanoparticles. Subsequently, the cells were irradiated with a dose of 6 Gy and returned to the incubator for another 2 h. Following irradiation, the cells were incubated with the DNA detection kit (γ-H2AX immunofluorescence method) purchased from Shanghai Biyuntian Biotechnology Co. Cell staining was observed using a confocal microscope.

### Immunogenic death evaluation

Calreticulin (CRT) exposure and HMGB1 release were analyzed using immunofluorescence. The 4T1 cells were plated in confocal dishes and incubated overnight. Then, the experimental groups were treated with different nanoparticles (Hf: 1 µg/mL, Mn: 5 µg/mL) and RT (6 Gy). The cells were fixed after 24 h of culture, permeabilized, and incubated with CRT and HMGB1 antibodies. Finally, the cells were incubated with FITC-labeled secondary antibody and nuclei were stained with DAPI. Images were acquired using confocal laser scanning microscope (CLSM). Extracellular ATP secretion was measured using an ELISA kit.

### DC maturation in vitro

After establishing the experimental groups (PBS, H, HM, HMG, RT, H + RT, HM + RT, and HMG + RT), 4T1 cells were inoculated into a six-well plates and incubated for 24 h. The culture medium was then replaced with medium containing different nanoparticles ( Hf: 1 µg/mL, Mn: 5 µg/mL), and the cells were further incubated for 6 h. Next, the cells designated for RT treatment were exposed to 6 Gy of radiation from an irradiator and incubation for 20 h.

Then, 1 mL of supernatant from the processed six-well plate was transferred to another six -well plate already seeded with DC cells and cultured for another 24 h. Subsequently, 1 mL of PBS containing CD80 (FITC), CD86 (APC), and CD11c (PE) antibodies was added. The plate was protected from light and incubated at 37 °C for 30 min. The cells were then collected to evaluate the maturation of the DC cells across different treatment groups using flow cytometry.

### Animal tumor model

Female BALB/c mice used in this study (4–6 weeks old) were purchased from the Laboratory Animal Services of Southern Medical University (Guangzhou, China). All animal care and in vivo studies were approved by the Animal Care and Use Committee of Southern Medical University and conducted in accordance with the ethical principles of the Ethics Committee for Animal Research (approval SMUL2023098). Subcutaneous tumor models in 4T1 cells were established by injecting 1 × 10^6^ 4T1 cells in PBS (100 µL) into the right-flank of female BALB/c mice. When the tumor volume reached 80–100 mm^3^, the tumor-bearing mice were randomly divided into groups for animal experiments.

### In vivo spectral CT imaging

Tumor-bearing mice were randomly divided into the saline group and the HMG group (*n* = 3 for each). A total of 20 µL of HMG nanoparticle aqueous solution (Hf: 1 mg/kg) or saline was administered into the tumor site via intra-tumoral injection. The mice were then scanned using a spectral CT before and 15 min after injection.

### In vivo MR imaging

Tumor-bearing mice were randomly divided into the saline group and the HMG group (*n* = 3 for each). The mice were scanned using a 3.0 T clinical magnetic resonance scanner with a T1-weighted sequence utilizing the following parameters: TR = 500.0 ms, TE = 14.8 ms, and slice thickness = 2 mm. For intra-tumoral injection, the scans were performed at different time points (0, 5, 15, 30, and 60 min) after the injecting HMG (2 mg/kg, Mn) or saline. For intra-venous injection, the mice were injected with 100 µL of HMG nanoparticle aqueous solution (4 mg/kg, Mn) or saline through the tail vein. The mice were then scanned at different time points after injection (0, 1, 3, 6, 12, and 24 h).

### Anti-tumor experiments in vivo

Unilateral tumor-bearing mice were randomly divided into eight groups (PBS, H, HM, HMG, RT, H + RT, HM + RT, and HMG + RT, *n* = 5). Subsequently, the mice received an intratumoral injection of 20 µL of saline or aqueous HMG nanoparticles (2 mg/kg, Mn). After 1 h, a local irradiation of 6 Gy was applied to the tumor region. Body weights and tumor sizes of the mice were recorded every other day. The mice were euthanized on day 14 of their treatment, and their blood, tumors, and major organs (heart, liver, spleen, lungs, and kidneys) were collected. Blood biochemistry, hematoxylin and eosin (H&E) staining, and immunohistochemistry tests were then conducted.

In the intravenous administration protocol, the mice received an injection of aqueous HMG nanoparticles (8 mg/kg, Mn) or saline via the tail vein, and irradiation was performed 6 h after the injection.

### Activation of cGAS-STING immune pathway

First, 1 × 10^6^ 4T1 cells in 100 µL of PBS solution were injected subcutaneously into the right dorsal side of each mouse. When the primary tumor volume reached 60–80 mm^3^, the mice were randomly assigned into the following eight groups: PBS, H, HM, HMG, RT, H + RT, HM + RT, and HMG + RT (*n* = 5). Subsequently, a 100 µL injection of either PBS or aqueous nanoparticle solution (8 mg/kg for Mn and 1.6 mg/kg for Hf) was administered via the tail vein. In the RT-treated group, the mouse tumor sites were exposed to 6 Gy of localized X-ray irradiation 6 h post-injection. Furthermore, a contralateral distant tumor model was established by injecting 100 µL of PBS solution containing 1 × 10^6^ 4T1 cells into the left hind dorsal region of the mice. Volumetric size measurement of the distant tumor and mice body weight were conducted daily starting on day 3 (considered as day 0). The mice were euthanized on day 14. Lymph nodes were collected for the extraction of lymphocytes, which were then co-incubated with CD80 (PE), CD86 (APC), and CD11c (PE-Cy7) antibodies for flow cytometry analysis. In addition, subcutaneous tumors from the right hind dorsal region of the mice were collected and tumor cells were extracted for western blotting (WB) assay to verify the expression of the cGAS-STING pathway-related proteins. Flow cytometry was also performed by co-incubation withCD4 (FITC), CD8 (PE), CD3 (APC), and CD45 (APC-Cy7) antibodies. ELISA assays were conducted to confirm serum expression levels of IFN-β, IFN-γ, TNF-α, and IL-6.

### Regulatory T cell analysis

To estimate the number of regulatory T cells (Tregs) in tumors, tumor cells were first extracted from mouse tumor tissues, subjected to red splitting and membrane-breaking treatments, and stained with fluorescent dye-labeled antibodies against CD45 (eFluor 450), CD3 (PE), CD4 (FITC), and Foxp3 (APC). Flow analysis was then carried out to evaluate the HMG NP mitigating effect on the immunosuppressive tumor microenvironment (TME) in mice.

### Statistical analysis

Statistical analysis was performed using GraphPad Prism 9. Student’s t-test was used to analyze the differences between two experimental groups, while one-way or two-way analysis was employed for multiple groups. Statistical significance was denoted by * for *p* < 0.05, ** for *p* < 0.01, *** for *p* < 0.001, and **** for *p* < 0.0001.

## Results and discussion

### Nanoparticle preparation and characterization

The construction process for HfO_2_@MnO_2_@GOx (HMG) is illustrated in Scheme 1. First, the MnO_2_ shell layer was decorated onto the HfO_2_ surface to form HfO_2_@MnO_2_ (HM) nanoparticles. Then GOx was loaded through an amide condensation reaction to prepare the final HMG nanoparticles.

**Fig. 1:**
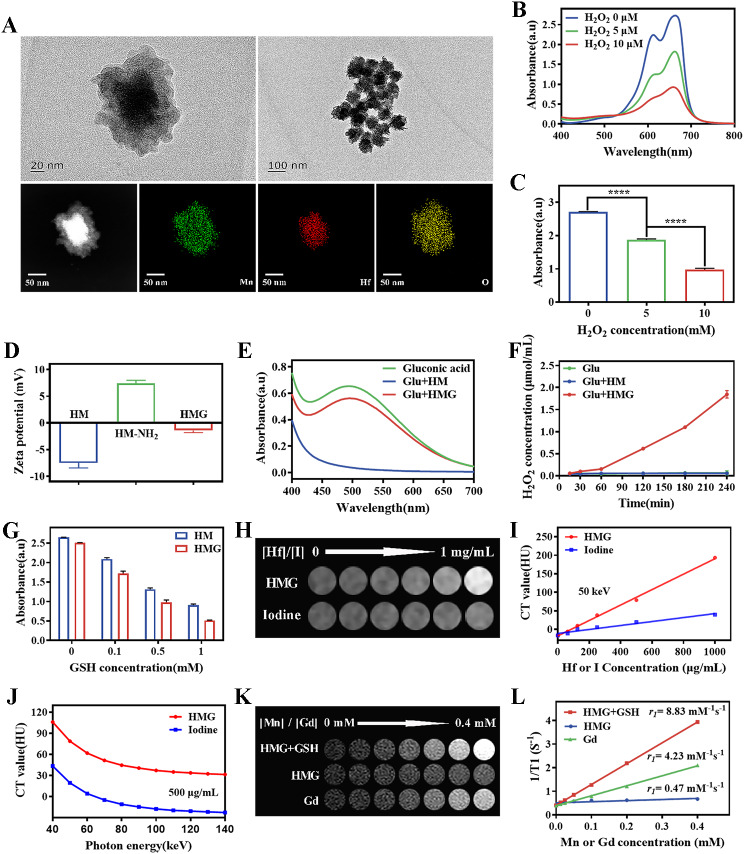
(**A**) The characterization of HfO_2_@MnO_2_ (HM) nanoparticles: TEM images and STEM-EDS elemental maps. (**B**-**C**) The ROS production ability of HM nanoparticles at different H_2_O_2_ concentrations measured by UV spectrophotometry. (**B**) Spectrograms in the range of 400–800 nm. (**C**) Comparison of absorption values at 665 nm for each group. (**D**) Changes in surface charge of nanoparticles during the amide reaction. Indirect reflection of GOx activity by UV spectrophotometric analysis of gluconic acid measurements (**E**) and by measurement of H_2_O_2_ content (**F**) in each group of solutions. (**G**) The ROS production ability of HM and HMG nanoparticles by oxidatively fading MB (665 nm) at different GSH concentrations (0, 0.1, 0.5, 1 mM). CT brightness (**H**) and density values (**I**) of HMG nanoparticles and iodine agent at different concentrations at 50 keV monoenergetic spectral CT. (**J**) CT values of HMG nanoparticles and iodine agent with increasing keV at Hf or I concentration of 500 µg/ mL. Magnetic resonance T1-weighted imaging (**K**) and T1 relaxation rates (**L**) of different materials (Gd-DTPA, HMG, HMG + GSH) with increasing concentrations of Mn or Gd. (****p < 0.0001)

Transmission electron microscopy maps and the STEM-EDS elemental map in Fig. 1A reveal that the HM nanoparticles were composed of Mn, Hf, and O elements. The specific morphology showed HfO_2_ nanoparticles as the core, with MnO_2_ forming thick-walled shells around it. This confirmed successful preparation of HM nanoparticles following the nanomaterial construction scheme. The Fenton-like reaction between Mn^2+^ and H_2_O_2_ produced a significant amount of hydroxyl radicals (-OH). The blue MB solution was oxidized to a colorless solution by the ROS present in the reaction system. Thus, its characteristic absorption peaks (light absorption value at 665 nm) were altered. The absorption spectrogram was significantly altered when H_2_O_2_ was present in the reaction system (Fig. 1B). Specifically, a noticeable reduction in absorption was found at 665 nm and was positively correlated with the H_2_O_2_ content (Fig. 1C). These results demonstrated that the constructed HM nanoparticles effectively increased the amount of ROS in the system with increasing H_2_O_2_ content.

Therefore, HM and GOx were combined to construct HMG nanoparticles based on the principle that GOx catalyzes glucose redox in the TME to produce more H_2_O_2_ followed by ROS cascade amplification. The attachment process of HM nanoparticles and GOx via amide synthesis is demonstrated in Fig. 1D, which confirmed the successful attachment of GOx through surface charge changes of products at various stages. Additionally, the successful incorporation of GOx enzymatic activity into HMG was further validated by detecting the two products (Glu and H_2_O_2_) of the reaction catalyzed by GOx. A distinct absorption peak characteristic of Glu was found in the Glu + HMG group, but not in the Glu + HM group (Fig. 1E). This suggests the presence of Glu in the Glu + HMG group solution. A similar trend was observed in Fig. 1F, where only the Glu + HMG group exhibited an increased level of H_2_O_2_ content. These findings demonstrated both the successful preparation of HMG nanoparticles and the effective catalytic performance of GOx within HMG nanoparticles. The HMG nanoparticles had good water solubility and stability in different solutions (Additional file 1: Fig. S1). The TME was characterized by high H_2_O_2_ and GSH levels and low pH condition, which had a significant impact on the therapeutic outcome. Nanoparticles that were responsive to these characteristics were more advantageous and promising for tumor therapy. Additional file 1: Fig. S2 and S3 confirm that acidic conditions, H_2_O_2_, and GSH were more conducive to the release of Mn^2+^ and O_2_ from HMG nanoparticles, suggesting that HMG nanoparticles were not only responsive to the TME, but also improved hypoxia to provide a suitable environment for tumor treatment.

Figure 1G further demonstrates that GSH influenced subsequent ROS generation by affecting the release Mn^2+^ from the nanoparticles. There was a concurrent decrease in the absorption value at 665 nm in the solution with an increase in GSH concentration, indicating an increase in the ROS level. Under identical conditions and barring variations between the HM and HMG nanoparticles, it was observed that the HMG nanoparticles consistently exhibited a superior capacity for ROS generation compared to the HM nanoparticles. These findings confirmed the enhanced ROS production capability of HMG nanoparticles.

The presence of the Hf element in HMG nanoparticles allowed for effective X-ray absorption, making them suitable for contrast-enhanced CT imaging. An in vitro CT imaging study was conducted using the state-of-art spectral CT. The results (Fig. 1H-J) revealed a gradual increase in CT density of the HMG nanoparticles as the material concentration increased. Furthermore, the CT values of both HMG nanoparticles and clinically used iodine contrast agent in monoenergetic images were studied using a wide range of photon energies (40–140 keV). The results (Additional file 1: Fig. S4) showed that the HMG nanoparticles exhibited superior CT imaging ability compared to the iodine agent in monoenergetic images, with a more pronounced difference in lower keV monoenergetic images. Therefore, it could be concluded that the HMG nanoparticles have a favorable spectral CT imaging performance.

Since Mn^2+^ is paramagnetic, it is suitable for magnetic resonance imaging (MRI) T1 contrast enhancement. The MR imaging ability of the HMG nanoparticles alone was minimal (Fig. 1K-L). In contrast, when HMG and GSH were simultaneously present, the T1-weighted MRI signals of the nanomaterial showed a significant increase with higher Mn concentrations in the nanoparticles. These signal intensities were even higher than those observed at the same concentration of clinically used gadolinium diamine. The T1 relaxation rate r_1_ value of each group also verified this phenomenon. The highest r_1_ value in the HMG + GSH group was 8.83 mM^-1^s^-1^. The results demonstrated excellent MR T1-weighted imaging and GSH responsive properties of HMG nanoparticles.

### Ferroptosis verification

**Fig. 2:**
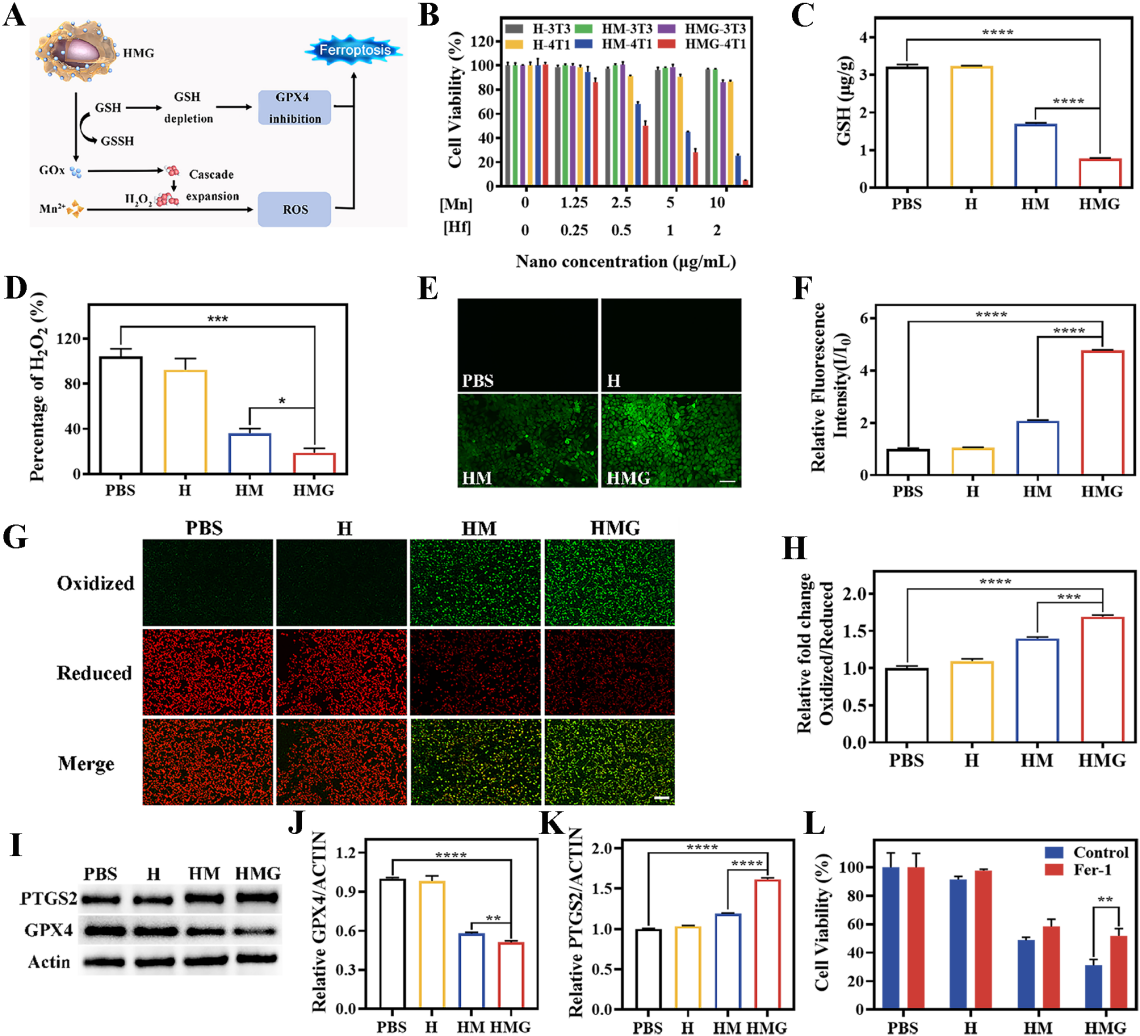
(**A**) Schematic diagram of ferroptosis. (**B**) Cell viability after incubation of 3T3 or 4T1 cells with different nanoparticles (H, HM, and HMG). (**C**) Intracellular GSH and (**D**) H_2_O_2_ content of 4T1 cells after co-incubation with different nanomaterials. ROS content observed and measured by inverted microscopy (**E**) and flow cytometry (**F**) after co-incubation of 4T1 cells with different nanomaterials. Fluorescence images (**G**) and flow analysis (**H**) of 4T1 cells treated with different nanoparticles (PBS, H, HM, and HMG) to assess the extent of intracellular lipid peroxidation using C11-BODIPY. The expression of intracellular GPX4 and PTGS2 proteins in 4T1 cells by after treatment with different nanoparticles by western blotting (**I**) and the relative expression of GPX4 (**J**) and PTGS2 (**K**) proteins in different treatment groups. (**L**) Cell viability after incubation of different groups of 4T1 cells with Fer-1 (ferroptosis inhibitor). (Scale bar = 100 μm; *p < 0.1, ** *p* < 0.01, *** *p* < 0.001, **** *p* < 0.0001)

The mechanism of HMG nanoparticles in the TME inducing tumor cells to undergo ferroptosis is illustrated in Fig. 2A.

First, the CCK-8 experiments were conducted to assess the toxic effects of various nanoparticles on both normal 3T3 mouse embryonic fibroblasts and 4T1 mouse breast cancer cells. The viability of the 3T3 cells remained above 80% at increasing concentrations of nanoparticles when exposed to different nanomaterials (Fig. 2B). This indicates that all three nanoparticles (H, HM, and HMG) exhibited no obvious cytotoxicity towards normal cells. Conversely, a substantial discrepancy in cytotoxicity was observed when cancerous 4T1 cells were subjected to different nanoparticles and concentrations. Notably, at Mn concentrations higher than 5 µg/mL (or 1 µg/mL for Hf) in the nanoparticles, both the HM and HMG nanoparticles demonstrated killing effects on the 4T1 cells, with the HMG group showing a more pronounced effect. This finding can be attributed to the higher concentrations of GSH and H_2_O_2_ contained in tumor cells, which facilitate the release of Mn^2+^ from the nanoparticles, leading to cell death by ROS via a Fenton-like reaction with H_2_O_2_. Owing to their functional GOx enezyme cascade, the HMG nanoparticles amplified the intracellular ROS levels, resulting in greater damage to the 4T1 cells. These findings are further demonstrated by the results presented in Fig. 2C-F, which confirmed that the HMG nanoparticles consumed the highest levels of GSH and H_2_O_2_ and generated the greatest amount of ROS in the 4T1 breast cancer cells.

The lipid peroxidation of polyunsaturated fatty acids, which is expressed abundantly on the cell membrane, is a crucial indicator of ferroptosis. The C11 BODIPY 581/591 lipid peroxidation probe was utilized to evaluate the extent of cellular lipid peroxidation in 4T1 cells to assess the occurrence of ferroptosis. The 4T1 cells in the HMG-treated group exhibited the highest levels of green fluorescence, suggesting the most pronounced lipid peroxidation and cellular ferroptosis (Fig. 2G-H). Consistent findings were obtained by analyzing the relative ratios of lipid peroxidation and reduced lipid measurements in 4T1 cells using flow cytometry analysis.

Next, WB was conducted to verify the expression of ferroptosis-related proteins (GPX4 and PTGS2) in 4T1 cells treated with various nanoparticles. The GSH-GPX4 pathway is prominent in regulating ferroptosis, and cells with GPX4 down-regulation exhibit increased sensitivity to ferroptosis. PTGS2 serves as a marker molecule for ferroptosis in vivo. Its expression up-regulation is potential to enhance ferroptosis. The expression of GPX4 and PTGS2 proteins in 4T1 cells co-incubated with H nanoparticles showed no significant changes compared to the control group (Fig. 2I-K). In contrast, GPX4 expression was down-regulated, while PTGS2 was up-regulated in 4T1 cells co-incubated with HM and HMG nanoparticles. These changes were most pronounced in the HMG group. Consequently, these results indicate that the HMG nanoparticles possess the highest ability to promote ferroptosis in 4T1 breast cancer cells.

To determine whether cell death induced by nanoparticles is mediated through ferroptosis, a feedback experiment was conducted in 4T1 cells using ferroptosis inhibitor Fer-1. Neither PBS nor the H group had any killing effect on 4T1 cells when treated with Fer-1 (Fig. 2L). However, the killing effect of HM and HMG nanoparticles on the cells was inhibited by Fer-1. Specifically, HMG inhibition of the killing effect in 4T1 cells increased from approximately 25–55%. The in vitro cellular experiments described above demonstrated that the HMG nanoparticles induced ferroptosis in breast cancer 4T1 cells.

### Radiosensitization and immune activation in vitro

**Fig. 3:**
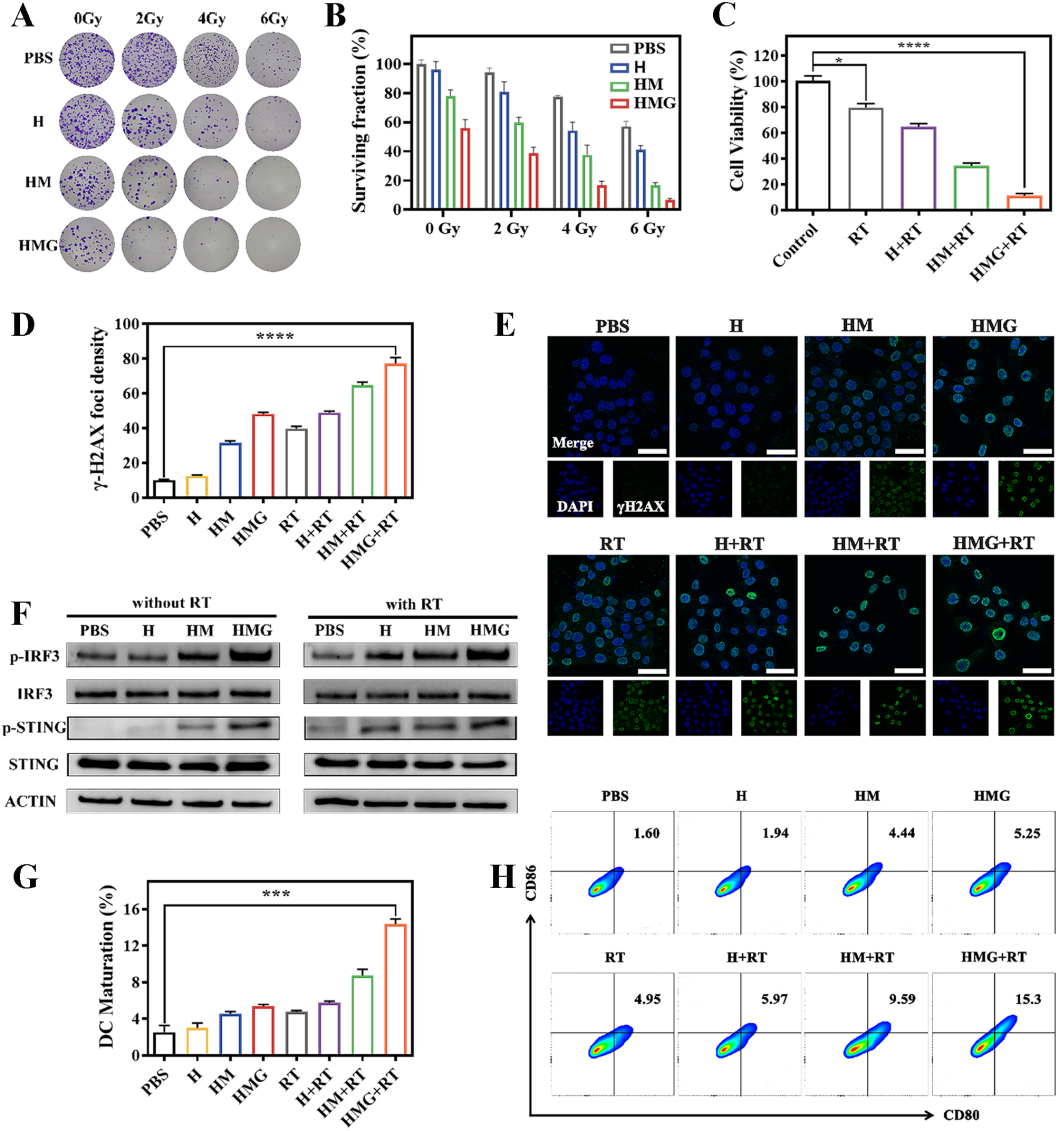
Optical diagrams (**A**) and counts (**B**) of colony formation of 4T1 cells after incubation with PBS or different nanoparticles (H, HM, and HMG) after exposure to different radiation doses (0, 2, 4, 6 Gy). (**C**) Cell viability of 4T1 cells after different treatments. Fluorescence intensity (**D**) and confocal fluorescence images (**E**) of DNA damage in 4T1 cells under different treatments. (**F**) Expression of cGAS-STING pathway-related proteins in 4T1 cells of different treatment groups. (**G**-**H**) The maturation of DC cells under different treatments assessed by flow cytometry. (Scale bar = 50 μm; ***p < 0.001, ****p < 0.0001)

Recent literature reported that ferroptosis has a close relationship with RT and plays a significant role in sensitizing tumors to radiation. RT primarily affects the proliferative capacity of cells, making it a long-term effect. To assess the ability of nanoparticles to sensitize 4T1 breast cancer cells to RT, the colony formation assay was conducted using varying radiation doses. The 4T1 cells incubated with HMG nanoparticles exhibited the lowest colony formation rate, following exposure to different radiation doses (Fig. 3A-B). In the HMG group, insignificant colony formation was observed when the radiation dose reached 6 Gy. Figure 3C depicts the CCK-8 assay results, which determined the cell survival rate of 4T1 cells co-incubated with various nanoparticles after the RT treatment. The results revealed that the HMG + RT group had the lowest cell survival rate of approximately 15%, which was significantly lower than that of the other groups. These findings confirmed that HMG nanoparticles had a more pronounced radiosensitization effect on 4T1 breast cancer cells than H and HM nanoparticles.

Cell radiosensitivity was achieved through the induction of DNA damage by RT. Immunofluorescence staining using the γ-H2AX marker was performed on 4T1 cells to reflect the extent of cellular DNA damage. The HMG + RT treatment group exhibited the highest level of green fluorescence, indicating the most severe DNA damage to breast cancer cells (Fig. 3D-E). This finding was consistent with the results of the colony formation experiment. The activating effect of damaged DNA on the cGAS-STING pathway was validated at the level of 4T1 cells. Figure 3F demonstrates that HMG and HMG + RT treatments on 4T1 cells caused the most significant up-regulation of the cGAS-STING pathway-related proteins. The in vitro results confirmed that HMG nanoparticles effectively mobilized this pathway to stimulate the immune response. Furthermore, in addition to causing DNA damage that activated the immune pathways, RT induced immunogenic cell death (ICD) activation of immunity in tumor cells [38]. CRT exposure, HMGB1 release, and ATP were characteristic of cells undergoing ICD. The most obvious green fluorescence of CRT was observed in 4T1 cells in the HMG + RT group, while green fluorescence of HMGB1 and ATP was the lowest among the groups, which demonstrated that the HMG + RT treatment group induced the formation of the most intense ICDs in the tumor cells (Additional file 1: Fig. S5). Damaged DNA and induced ICDs activate the immunity to induce DC cell maturation. In vitro, flow cytometry analysis was conducted to compare the induction of DC cell maturation among various treatment groups. The HMG + RT group had the most pronounced impact on DC cell maturation, highlighting the ability of HMG nanoparticles to sensitize RT and optimize DC cell maturation for immune response activation (Fig. 3G-H).

### Imaging and anti-tumor efficiency in vivo (intratumoral injections)

HfO_2_ was employed as a radiosensitizer via intratumoral injection in clinic. Consequently, in vivo experiments were initiated using HMG nanoparticles with HfO_2_ as the construct core by intratumoral injection. Significantly elevated CT density at the mouse tumor site was only found in the 50 keV monoenergetic image after intratumoral injection of HMG nanoparticles (Additional file 1: Fig. S6). This result confirmed the CT imaging contrast enhancement performance of HMG nanoparticles in vivo and highlighted the advantages of spectral CT in breast cancer diagnosis. Specifically, it indicated that utilizing the unique monoenergetic images reconstructed from spectral CT improved the sensitivity of breast cancer diagnosis by employing lower keV CT imaging.

**Fig. 4:**
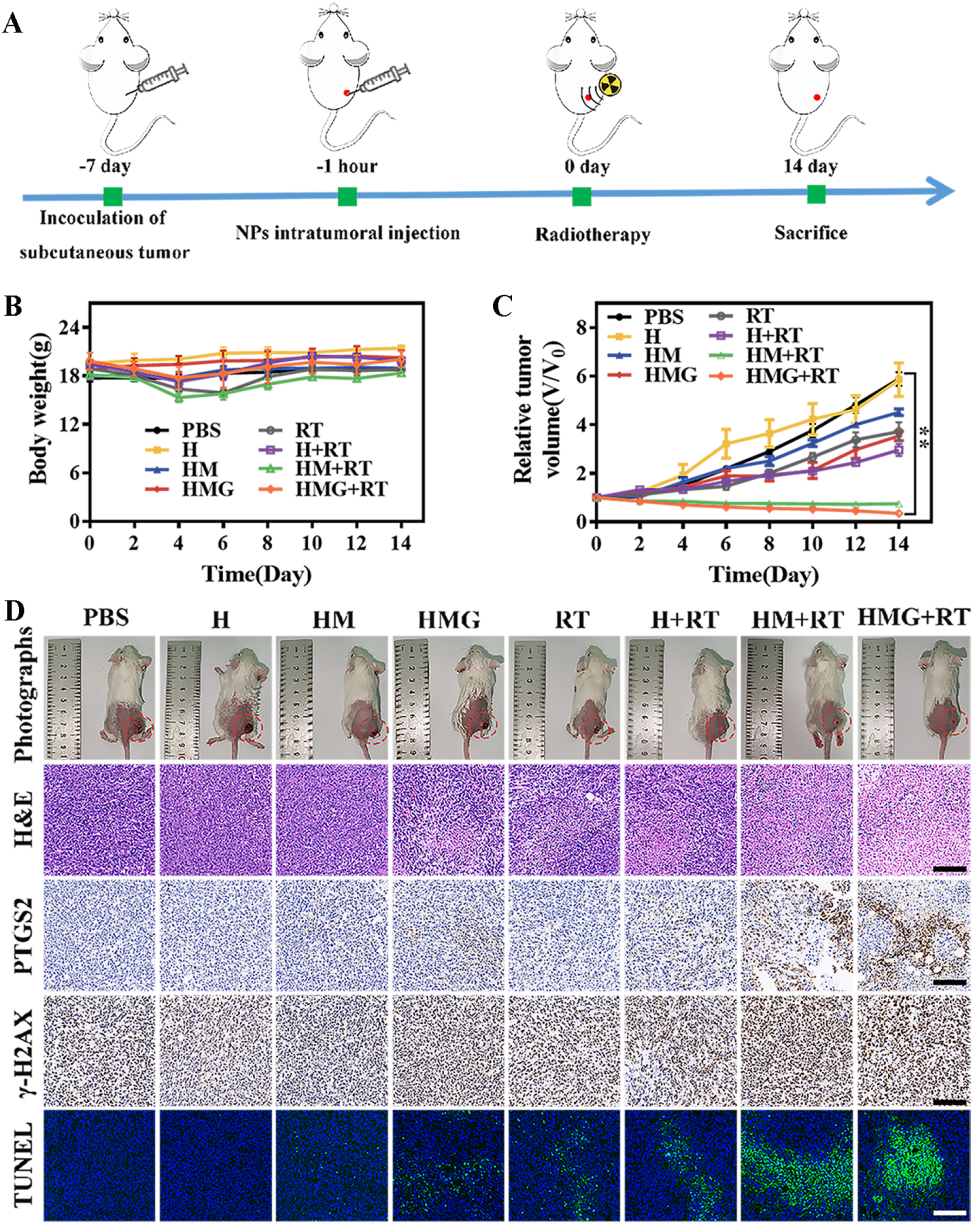
(**A**) Illustration of the treatment schedule used to assess the impact of intratumorally injected nanoparticles on primary tumors. Body weight (**B**) and tumor volume (**C**) changes of mice in each treatment group. (**D**) Photograph, H&E staining, immunochemical staining (PTGS2, γ-H2AX), and TUNEL staining in tumor-bearing mice after 14 days of treatment. (Scale bar = 200 μm; **p < 0.01)

Additional file 1: Fig. S7 illustrated the magnetic resonance T1-weighted imaging performance of HMG nanoparticles when injected at the tumor site. These results demonstrated that the extent of the signal within the tumor region exhibited a centrifuge enhancement pattern over time. This phenomenon was attributed to the interaction between HMG and GSH in the TME, leading to the gradual release of paramagnetic Mn^2+^. This finding further confirmed the GSH responsiveness of HMG nanoparticles in vivo. The procedure for the tumor suppression assay (intratumoral injection) is represented in Fig. 4A. Mouse body weights in each group did not exhibit any significant changes throughout the treatment period (Fig. 4B). Moreover, the HMG + RT group displayed the most notable tumor inhibition, with an inhibition rate approaching 67%, in comparison to the other groups (Fig. 4C). This result was further confirmed by mouse optical photographs (Fig. 4D). The immunohistochemical results revealed that the mouse tumor cells in the HMG + RT group exhibited the highest expression of PTGS2 and γ-H2AX, indicating a more prominent ferroptosis and cellular DNA damage. Additionally, H&E and TUNEL staining results further demonstrated that the HMG nanoparticles enhanced RT sensitivity through ferroptosis induction to effectively suppress breast cancer growth. Blood biochemistry and H&E staining results (Additional file 1: Fig. S8) of major organs in all treated mice showed no obvious abnormalities, verifying the favorable biosafety of the HMG nanoparticles.

### Imaging and anti-tumor efficiency in vivo (tail vein injection)

**Fig. 5:**
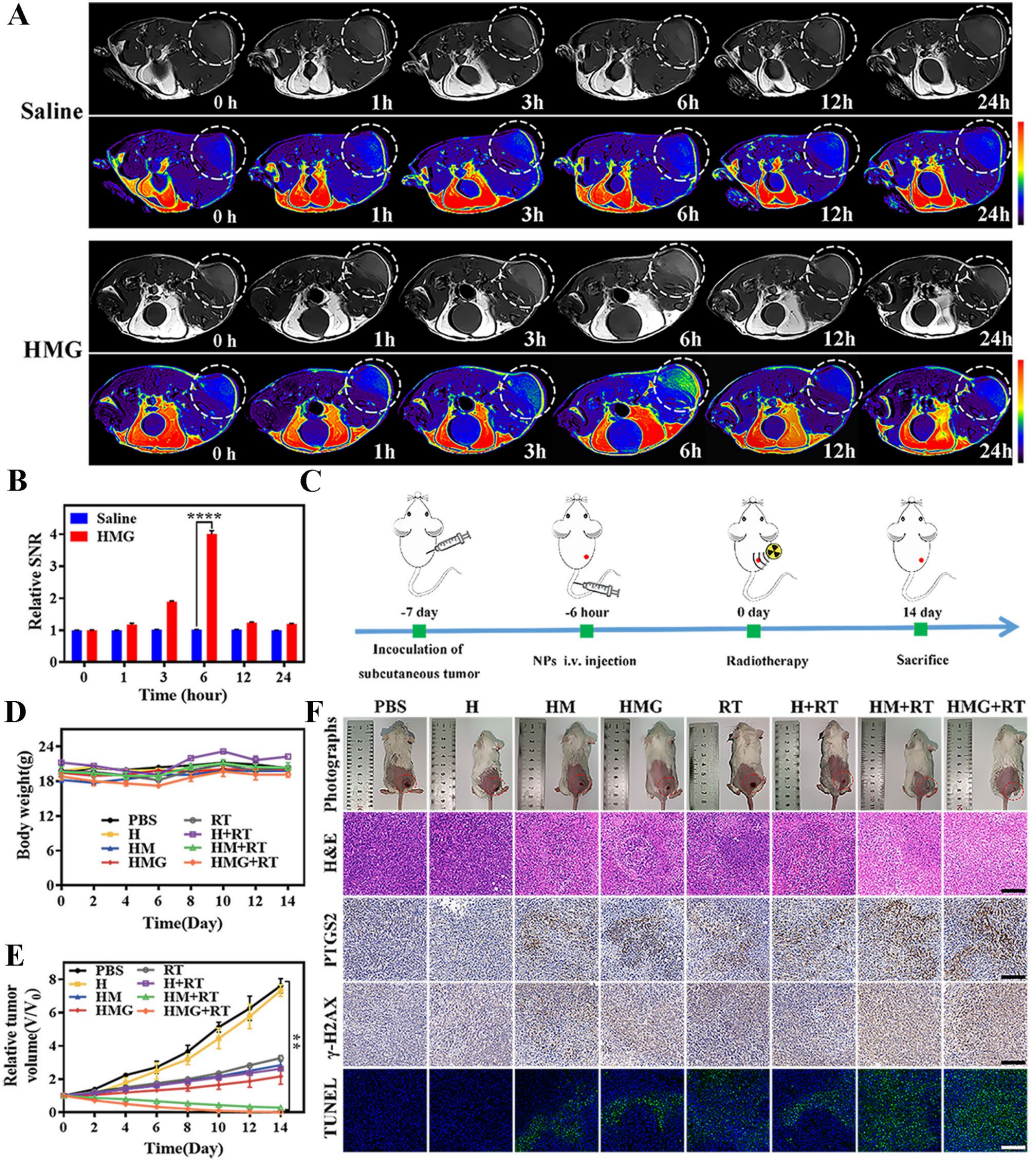
Magnetic resonance T1-weighted images (**A**) and relative signal-to-noise ratio (**B**) of mice before and after intravenous injection of saline or HMG nanoparticle solution. (**C**) Illustration of the treatment schedule used to assess the impact of i.v. injected NPs on primary tumors. (**D**) Body weight and (**E**) tumor volume changes in each treatment group. (**F**) Photograph, H&E staining, immunochemical staining (PTGS2, γ-H2AX), and TUNEL staining in mice after 14 days of treatment. (Scale bar = 200 μm; **p < 0.01, ****p < 0.0001)

Intratumoral drug delivery alone poses challenges in tumor therapy due to complexities in the procedural aspect and uneven drug distribution within the tumor tissue. To overcome these drawbacks, intravenous delivery protocols were explored, aiming to enhance the therapeutic effects and application prospects of HMG nanoparticles in tumor therapy. The HMG nanoparticles were intravenously injected (Fig. 5A-B), demonstrating increasing enrichment at the tumor site over time on MRI, with the most noticeable accumulation observed 6 h post-injection. This time point also served as a suitable window for subsequent antitumor treatment (Fig. 5C).

Figure 5D and Additional file 1: Fig. S9 illustrate the biological safety of HMG nanoparticles via tail vein injections in mice. Compared to other treatment groups, the HMG + RT group demonstrated the most significant tumor suppression (Fig. 5E), with a higher level of ferroptosis and DNA damage found in the mouse tumor tissues (Fig. 5F). These findings strongly suggested that the HMG nanoparticles, whether administered via intratumoral or venous injection, possess effective anti-tumor properties in tumor-bearing mice.

### Immune response activation in vivo

**Fig. 6:**
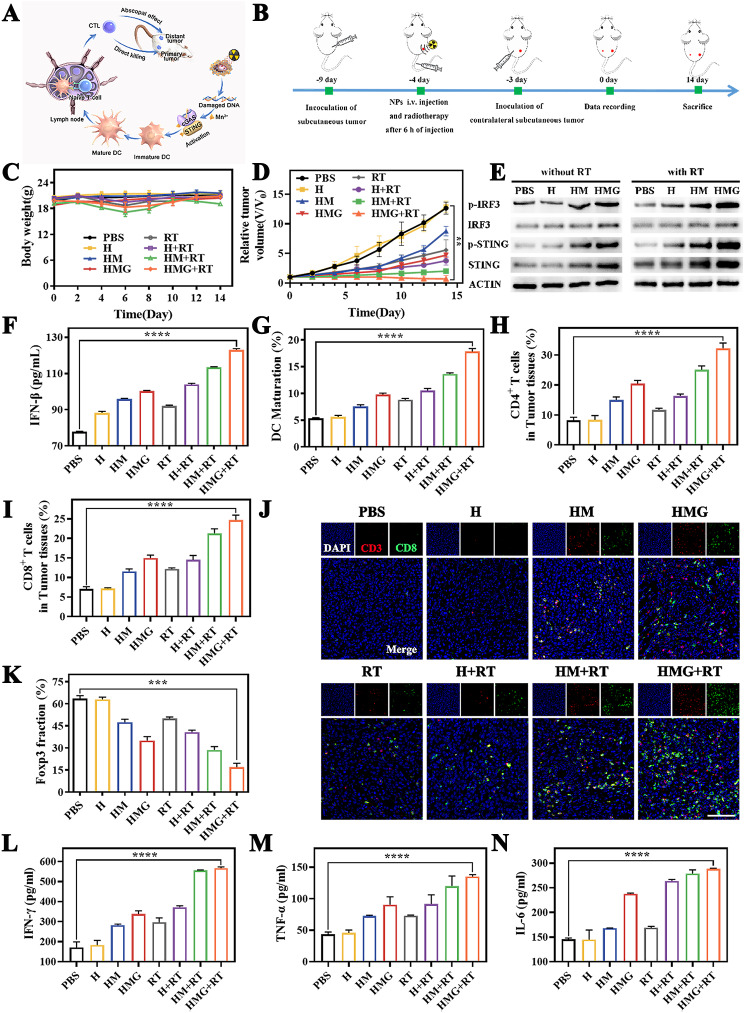
Activation of the immune response in vivo. (**A**) Schematic diagram of immune response in vivo. (**B**) Schematic illustration showing the animal experiment design. Changes in body weight (**C**) and volume of contralateral metastatic tumors (**D**) during treatment. (**E**) Expression of cGAS-STING pathway-related proteins in tumortissues. (**F**) Levels of IFN-β in the serum. (**G**) Maturation of DC cells in the lymph nodes of mice after treatment. (**H**-**I**) Proportion of CD4^+^ and CD8^+^ T cells in tumor tissues after treatment. (**J**) Immunofluorescence images of tumor sections for CD8^+^ T cells stained with CD3 (red), CD8 (green), and nuclear (blue). (**K**) The quantification of CD4^+^ FOXP3^+^ (Treg) in tumor. (L-N) Levels of IFN-β, TNF-α, and IL-6 at tumor sites in mice. (Scale bars: 100 μm, **p < 0.01, ***p < 0.001, ****p < 0.0001)

A schematic diagram of immune response in vivo is represented in Fig. 6A. A contralateral distant tumor model (Fig. 6B) was constructed to confirm the immune response induction by HMG + RT in tumor-bearing mice. Mouse body weights in different groups exhibited minimal changes (Fig. 6C). Notably, the HMG + RT group had the most significant inhibitory effect on distant tumors in mice with the tumor inhibition rate of up to 98.7% (Fig. 6D). It is noteworthy that this tumor regression was induced by a systemic anti-tumor immune response in mice through the combined effect of HMG nanoparticles and RT.

To further verify the immune activation effect of HMG nanoparticles combined with RT on distant tumors, the expression of specific proteins (STING, p-STING, IRF3, and p-IRF3) related to the cGAS-STING immune signaling pathway was explored using western blotting. The expression of all relevant proteins in the HMG + RT group exhibited significant up-regulation (Fig. 6E). The p-STING/STING and p-IRF3/IRF3 ratios after HMG treatment were higher than those of other groups, further confirming the potent activation of the cGAS-STING pathway by HMG nanoparticles (Additional file 1: Fig. S10). The downstream IFN-β pathway signal was also significantly up-regulated (Fig. 6F). These results demonstrated that the HMG nanoparticles had a substantial promoting effect on the cGAS-STING signaling pathway during RT.

Furthermore, DC maturation in the mouse lymph nodes was assessed using flow cytometry. The HMG + RT group induced the highest level of DC cell maturation (Fig. 5G and Additional file 1: Fig. S11). Notably, both CD4^+^ and CD8^+^ T cells are key T lymphocytes in the immune response to antitumor therapy. Compared to other treatment groups, mice in the HMG + RT group had the highest proportion of CD4^+^ and CD8^+^ T cells at the tumor site (Fig. 6H-I and Additional file 1: Fig. S12). Immunofluorescence images showed that a large number of CD3^+^/CD8^+^ T cells were aggregated in the tumor region of mice in the HMG + RT treatment group (Fig. 6J), indicating that tumor-specific immunity was activated effectively. In addition, the immunosuppressive effect of HMG nanoparticles on TME was also explored. The main function of Tregs is to suppress various immune responses, and Foxp3 is a key Treg transcription factor [39]. Figure 6K and Additional file 1: Fig. S13 demonstrate that HMG + RT significantly reduced Tregs, thereby alleviating TME immunosuppression. Additionally, the inflammatory cytokine levels (IFN-γ, TNF-α, and IL-6) in tumor tissues were measured using ELISA experiments. The HMG + RT group induced the highest secretion levels of IFN-γ, TNF-α, and IL-6 among all groups (Fig. 6K-M). Taken together, these results indicated that the combination of HMG nanoparticles and RT optimized the activation of the anti-tumor immune response in vivo.

## Conclusion

In this study, HfO_2_@MnO_2_@GOx (HMG) nanomaterials were successfully constructed using HfO_2_ as the core material, modifying the outer layer with MnO_2_ shell, and coupling GOx via amide condensation reaction. The in vitro and in vivo experiments demonstrated that HMG nanoparticles exhibited GSH-responsive properties in the tumor cell microenvironment. By depleting intracellular GSH, tumor cells inhibited the GPX4 signaling pathway, promoting ferroptosis to sensitize RT. Furthermore, the Fenton-like reaction between MnO_2_ and GOx led to the cascading amplification of ROS, inducing ferroptosis and sensitizing RT. Additionally, HfO_2_ enhanced RT by increasing radiation deposition in the tumor area. Consequently, damaged DNA in tumor cells and Mn^2+^ in the cytoplasm activated the cGAS-STING immune signaling pathway to evoke systemic anti-tumor immune response. These HfO_2_@MnO_2_@GOx nanomaterials can be utilized for ferroptosis induction to sensitize RT combined with immunotherapy for breast cancer while guided by spectral CT and MR dual-modality imaging, showing great promise as nanoplatforms for tumor diagnosis and treatment.

### Electronic supplementary material

Below is the link to the electronic supplementary material.

Supplementary Material 1

## Data Availability

All data generated and analyzed during this research are available on reasonable request from the authors.

## References

[1] Siegel R, Giaquinto A, Jemal A (2024). Cancer statistics, 2024. Cancer J Clin.

[2] Giaquinto A, Sung H, Miller K, Kramer J, Newman L, Minihan A, Jemal A, Siegel R (2022). Breast Cancer statistics, 2022. Cancer J Clin.

[3] Wheeler S, Rocque G, Basch E (2024). Benefits of breast Cancer screening and treatment on Mortality. JAMA.

[4] Caswell-Jin J, Sun L, Munoz D, Lu Y, Li Y, Huang H, Hampton J, Song J, Jayasekera J, Schechter C (2024). Analysis of breast Cancer mortality in the US-1975 to 2019. JAMA.

[5] Cao W, Jin M, Zhou W, Yang K, Cheng Y, Chen J, Cao G, Xiong M, Chen B. Forefronts and hotspots evolution of the nanomaterial application in anti-tumor immunotherapy: a scientometric analysis. J Nanobiotechnol 2024, 22. 10.1186/s12951-023-02278-3 PMC10788038 38218872

[6] Zhang X, Li S, Malik I, Do M, Ji L, Chou C, Shi W, Capistrano K, Zhang J, Hsu T (2023). Reprogramming tumour-associated macrophages to outcompete cancer cells. Nature.

[7] Swain S, Shastry M, Hamilton E (2023). Targeting HER2-positive breast cancer: advances and future directions. Nat Rev Drug Discovery.

[8] Dosta P, Cryer A, Dion M, Shiraishi T, Langston S, Lok D, Wang J, Harrison S, Hatten T, Ganno M (2023). Investigation of the enhanced antitumour potency of STING agonist after conjugation to polymer nanoparticles. Nat Nanotechnol.

[9] Zhao Y, Simon M, Seluanov A, Gorbunova V (2023). DNA damage and repair in age-related inflammation. Nat Rev Immunol.

[10] Kang N, Son S, Min S, Hong H, Kim C, An J, Kim J, Kang H (2023). Stimuli-responsive ferroptosis for cancer therapy. Chem Soc Rev.

[11] Liu D, Liang S, Ma K, Meng Q, Li X, Wei J, Zhou M, Yun K, Pan Y, Rao L et al. Tumor Microenvironment-Responsive nanoparticles amplifying STING Signaling Pathway for Cancer Immunotherapy. Adv Mater 2023:e2304845. 10.1002/adma.202304845 37723642

[12] Hopfner K, Hornung V (2020). Molecular mechanisms and cellular functions of cGAS-STING signalling. Nat Rev Mol Cell Biol.

[13] Concannon K, Morris B, Gay C, Byers L (2023). Combining targeted DNA repair inhibition and immune-oncology approaches for enhanced tumor control. Mol Cell.

[14] Cho M, Kumar R, Lin C, Boyer J, Shahir J, Fagan-Solis K, Simpson D, Fan C, Foster C, Goddard A (2024). MRE11 liberates cGAS from nucleosome sequestration during tumorigenesis. Nature.

[15] Zhang K, Qi C, Cai K (2023). Manganese-based Tumor Immunotherapy. Adv Mater.

[16] Cao Y, Ding S, Hu Y, Zeng L, Zhou J, Lin L, Zhang X, Ma Q, Cai R, Zhang Y et al. An Immunocompetent Hafnium Oxide-based STING nanoagonist for Cancer Radio-Immunotherapy. ACS nano 2024. 10.1021/acsnano.3c09293 38193384

[17] Yi L, Jiang X, Zhou Z, Xiong W, Xue F, Liu Y, Xu H, Fan B, Li Y, Shen J. A hybrid Nanoadjuvant simultaneously depresses PD-L1/TGF-β1 and activates cGAS-STING pathway to overcome radio-immunotherapy resistance. Adv Mater 2024:e2304328. 10.1002/adma.202304328 38229577

[18] Groelly F, Fawkes M, Dagg R, Blackford A, Tarsounas M (2023). Targeting DNA damage response pathways in cancer. Nat Rev Cancer.

[19] McLaughlin M, Patin E, Pedersen M, Wilkins A, Dillon M, Melcher A, Harrington K (2020). Inflammatory microenvironment remodelling by tumour cells after radiotherapy. Nat Rev Cancer.

[20] Galluzzi L, Aryankalayil M, Coleman C, Formenti S (2023). Emerging evidence for adapting radiotherapy to immunotherapy. Nat Reviews Clin Oncol.

[21] Cytlak U, Dyer D, Honeychurch J, Williams K, Travis M, Illidge T (2022). Immunomodulation by radiotherapy in tumour control and normal tissue toxicity. Nat Rev Immunol.

[22] Dong Q, Xue T, Yan H, Liu F, Liu R, Zhang K, Chong Y, Du J, Zhang H (2023). Radiotherapy combined with nano-biomaterials for cancer radio-immunotherapy. J Nanobiotechnol.

[23] Zhang J, Yang M, Fan X, Zhu M, Yin Y, Li H, Chen J, Qin S, Zhang H, Zhang K, Yu F (2022). Biomimetic radiosensitizers unlock radiogenetics for local interstitial radiotherapy to activate systematic immune responses and resist tumor metastasis. J Nanobiotechnol.

[24] Wu Y, Song Y, Wang R, Wang T (2023). Molecular mechanisms of tumor resistance to radiotherapy. Mol Cancer.

[25] Lei G, Mao C, Yan Y, Zhuang L, Gan B (2021). Ferroptosis, radiotherapy, and combination therapeutic strategies. Protein Cell.

[26] Zeng L, Ding S, Cao Y, Li C, Zhao B, Ma Z, Zhou J, Hu Y, Zhang X, Yang Y (2023). A MOF-Based potent ferroptosis inducer for enhanced Radiotherapy of Triple negative breast Cancer. ACS Nano.

[27] Chen X, Kang R, Kroemer G, Tang D (2021). Broadening horizons: the role of ferroptosis in cancer. Nat Reviews Clin Oncol.

[28] Xiong Y, Xiao C, Li Z, Yang X (2021). Engineering nanomedicine for glutathione depletion-augmented cancer therapy. Chem Soc Rev.

[29] Stockwell B (2022). Ferroptosis turns 10: emerging mechanisms, physiological functions, and therapeutic applications. Cell.

[30] Wei M, Bai J, Shen X, Lou K, Gao Y, Lv R, Wang P, Liu X, Zhang G (2023). Glutathione-Exhausting Nanoprobes for NIR-II fluorescence imaging-guided surgery and boosting Radiation Therapy Efficacy via Ferroptosis in breast Cancer. ACS Nano.

[31] Huang C, Liu Z, Chen M, Du L, Liu C, Wang S, Zheng Y, Liu W (2021). Tumor-derived biomimetic nanozyme with immune evasion ability for synergistically enhanced low dose radiotherapy. J Nanobiotechnol.

[32] Ding S, Chen L, Liao J, Huo Q, Wang Q, Tian G, Yin W (2023). Harnessing hafnium-based nanomaterials for Cancer diagnosis and therapy. Small.

[33] Skrodzki D, Molinaro M, Brown R, Moitra P, Pan D (2024). Synthesis and bioapplication of emerging nanomaterials of Hafnium. ACS Nano.

[34] Bonvalot S, Rutkowski P, Thariat J, Carrère S, Ducassou A, Sunyach M, Agoston P, Hong A, Mervoyer A, Rastrelli M (2019). NBTXR3, a first-in-class radioenhancer hafnium oxide nanoparticle, plus radiotherapy versus radiotherapy alone in patients with locally advanced soft-tissue sarcoma (Act.In.Sarc): a multicentre, phase 2–3, randomised, controlled trial. Lancet Oncol.

[35] Espenel S, Chargari C, Blanchard P, Bockel S, Morel D, Rivera S, Levy A, Deutsch E (2022). Practice changing data and emerging concepts from recent radiation therapy randomised clinical trials. Eur J Cancer.

[36] Zhou G, Li M (2022). Near-Infrared-II Plasmonic Trienzyme-Integrated Metal-Organic frameworks with high-efficiency enzyme cascades for synergistic Trimodal Oncotherapy. Adv Mater.

[37] Chen Y, Li H, Hou B, Wu A, Wu W, Li C, Wang H, Chen D, Wang X (2024). NaYF:Yb/Er@Mn O @GOX nanocomposite for Upconversion fluorescence imaging and synergistic Cascade Cancer Therapy by apoptosis and Ferroptosis. Small.

[38] Zhou M, Liang S, Liu D, Ma K, Yun K, Yao J, Peng Y, Hai L, Zhang Q, Wang Z (2023). Manganese-enriched zinc Peroxide Functional nanoparticles for Potentiating Cancer Immunotherapy. Nano Lett.

[39] Zhang W, Leng F, Wang X, Ramirez R, Park J, Benoist C, Hur S (2023). FOXP3 recognizes microsatellites and bridges DNA through multimerization. Nature.

