# Supplementary material for: Radiotherapy-sensitized cancer immunotherapy via cGAS-STING immune pathway by activatable nanocascade reaction
Source: J Nanobiotechnology. 2024 May 9;22:234. doi: 10.1186/s12951-024-02502-8 (PMC11080188; doi:10.1186/s12951-024-02502-8)
Supplement: Supplementary file 1 — Supplementary Material 1 [file 12951_2024_2502_MOESM1_ESM.docx]

**Additional file 1**

**Radiotherapy-sensitized Cancer Immunotherapy via cGAS-STING Immune Pathway by Activatable Nanocascade Reaction**

**Honglei Hu^1,3‡^, Shuting Zheng^1,2‡^, Chenxi He^1,2^, Yinfei Zheng^1,2^, Qiming Wei^4^, Siwen Chen^2^, Zede Wu^1,2^, Yikai Xu^1*^, Bingxia Zhao^2*^, and Chenggong Yan^1*^**


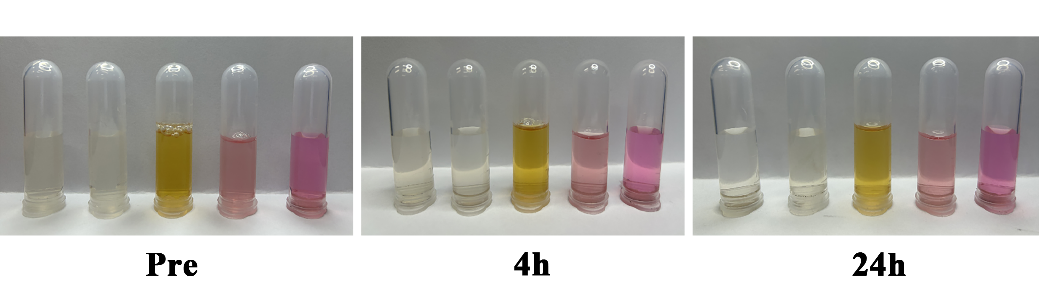


**Fig. S1.** Digital photographs of HMG nanoparticles after dispersion in different solutions for different times (from left to right: pure water, PBS, FBS, DMEM and DMEM with 10% FBS).


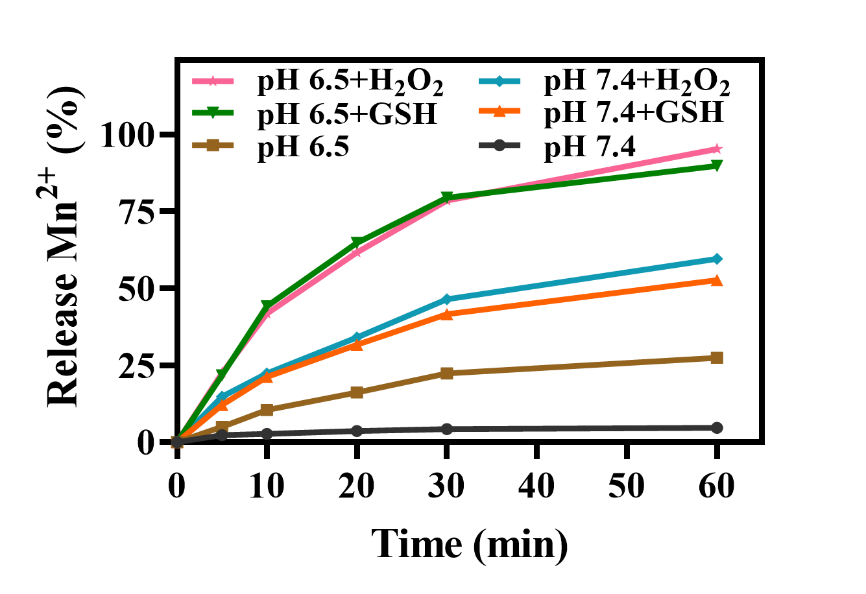


**Fig. S2.** Trends of Mn^2+^ release of HMG nanoparticles under different conditions (pH, H_2_O_2_ and GSH).


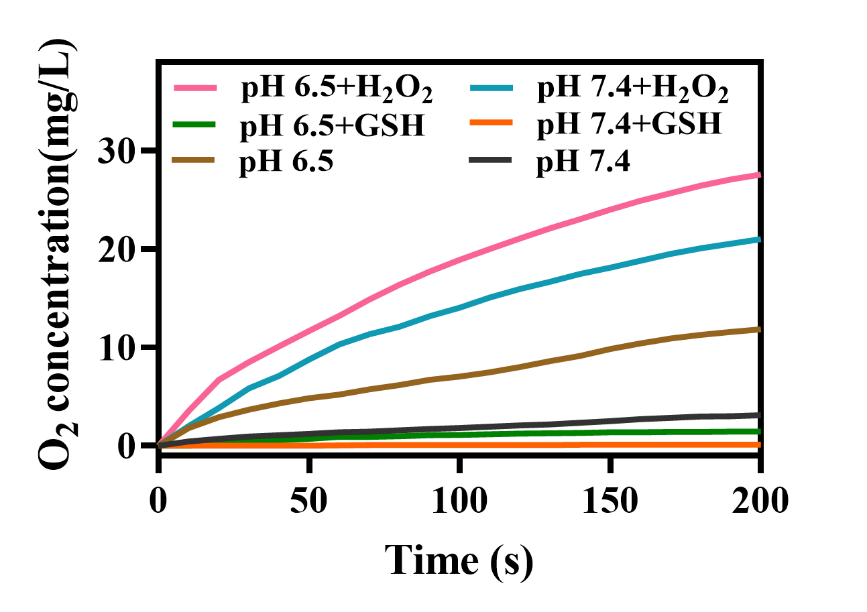


**Fig. S3.** O_2_ release time curve of HMG nanoparticles under different conditions (pH, H_2_O_2_ and GSH).


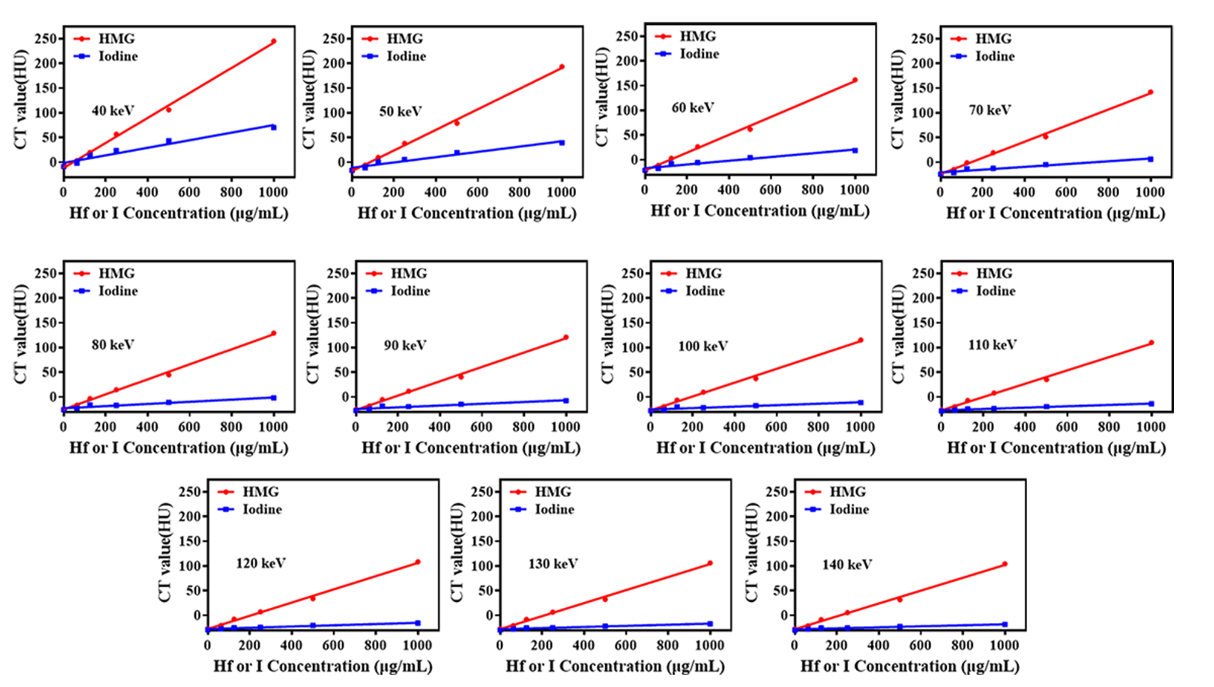


**Fig. S4.** The CT values of HMG nanoparticles and Iodine with increasing concentration under different keV conditions.


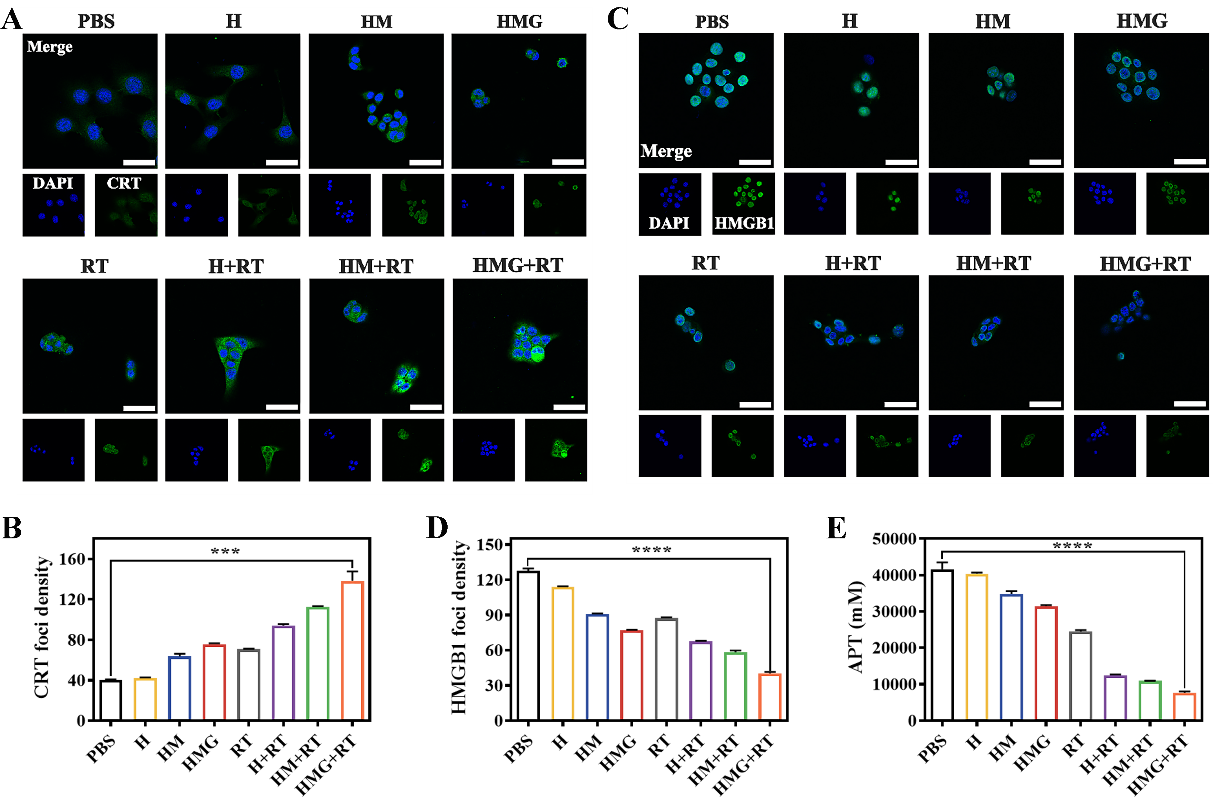


**Fig. S5.** Confocal fluorescence images (A) and foci intensity (B) of CRT in 4T1 cells under different treatments. Confocal fluorescence images (C) and foci intensity (D) of CRT in 4T1 cells under different treatments. (E) APT level in 4T1 cells under different treatments. (Scale bar = 50 μm; ****p* < 0.001, *****p* < 0.0001)


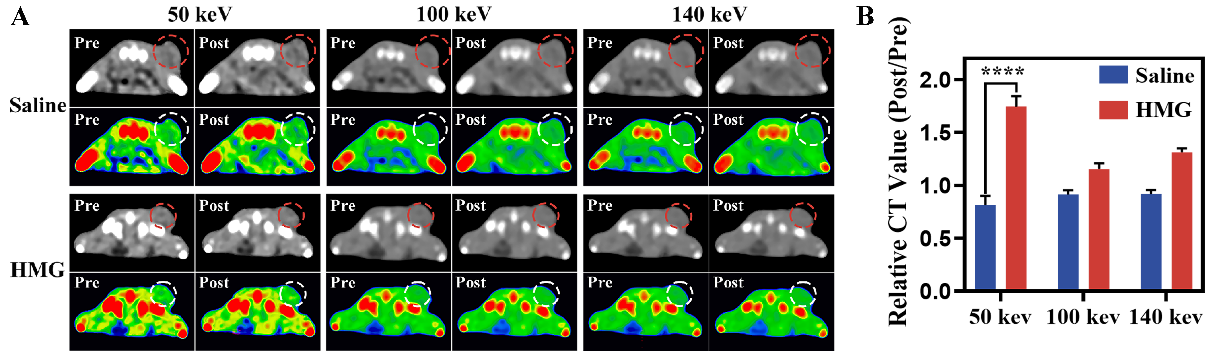


**Fig. S6.** (A) Spectral CT images (50, 100, and 140 keV) of mice before and after intratumoral injection of saline or HMG nanoparticle solution. (B) The relative CT value (post/pre) of the injection region at different keV. (*****p* < 0.0001)


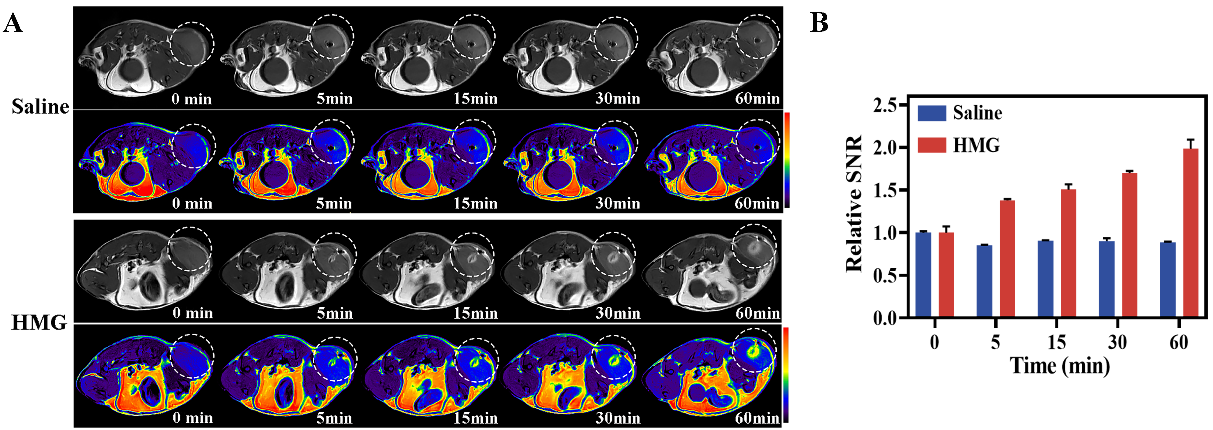


**Fig. S7.** Magnetic resonance T1-weighted images (A) and relative signal-to-noise ratio (B) of mice before and after intratumoral injection of saline or HMG nanoparticle solution.


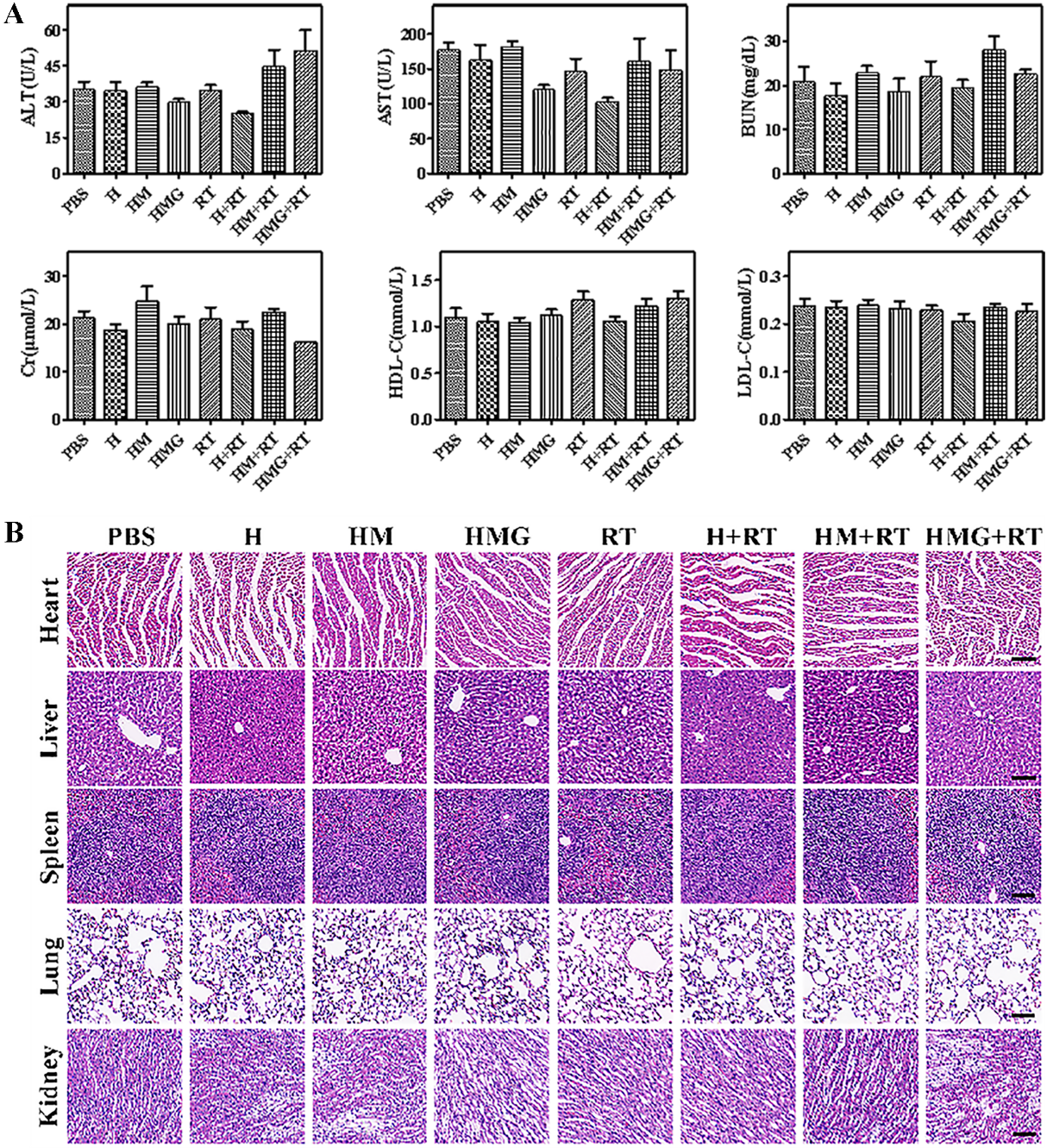


**Fig. S8.** (A) Blood biochemical results and (B) H&E staining of major organs of mice in each treatment group at day 14 post-treatment by intratumoral injection. (Scale bar = 200 μm)


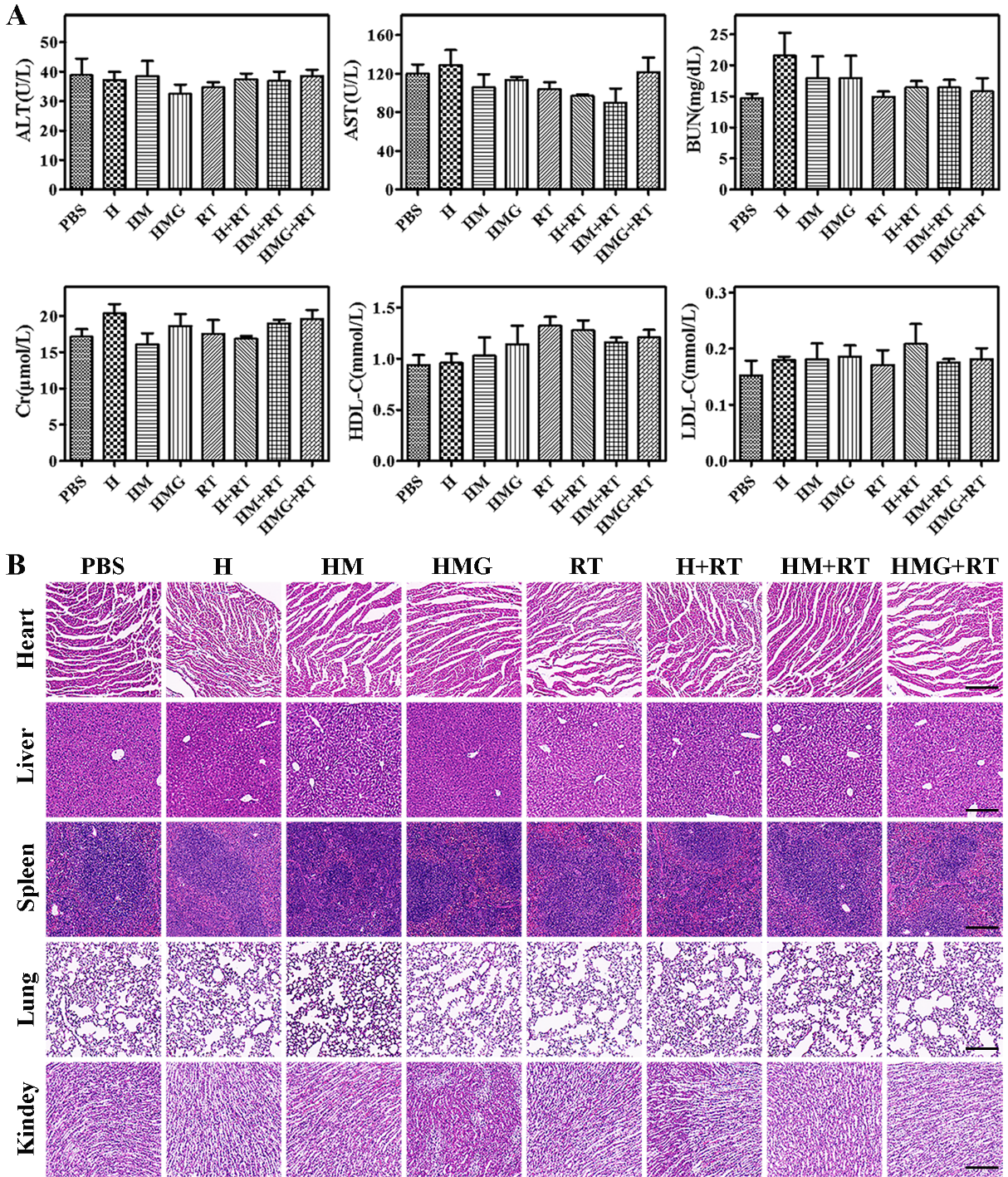


**Fig. S9.** (A) Blood biochemical results and (B) H&E staining of major organs of mice in each treatment group at day 14 post-treatment by tail vein administration. (Scale bar = 200 μm)


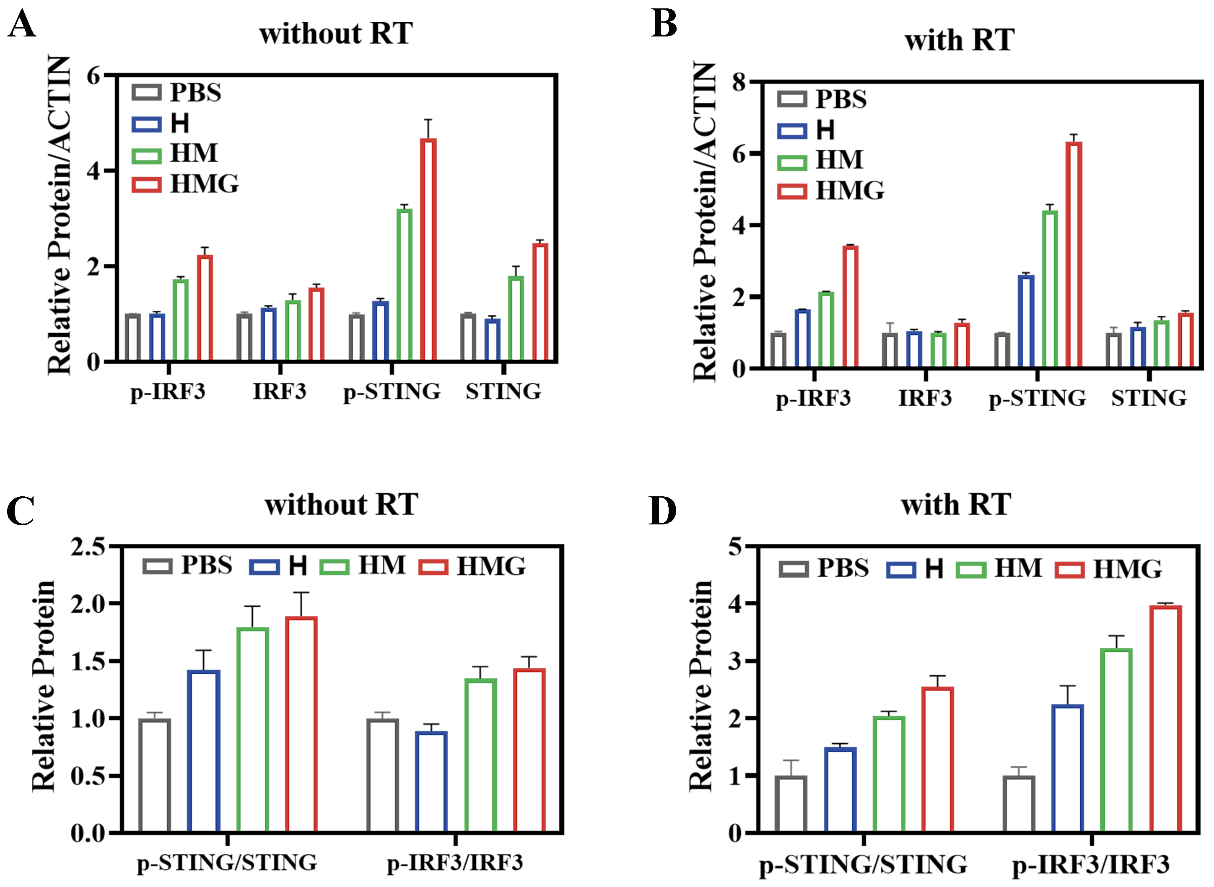


**Fig. S10.** (A-B) Relative expression of cGAS-STING pathway-related proteins (STING, p-STING, IRF3, p-IRF3) in mouse tumor cells. (C-D) The relative expression of p-STING/STING and p-IRF3/IRF3 proteins in different treatment groups.


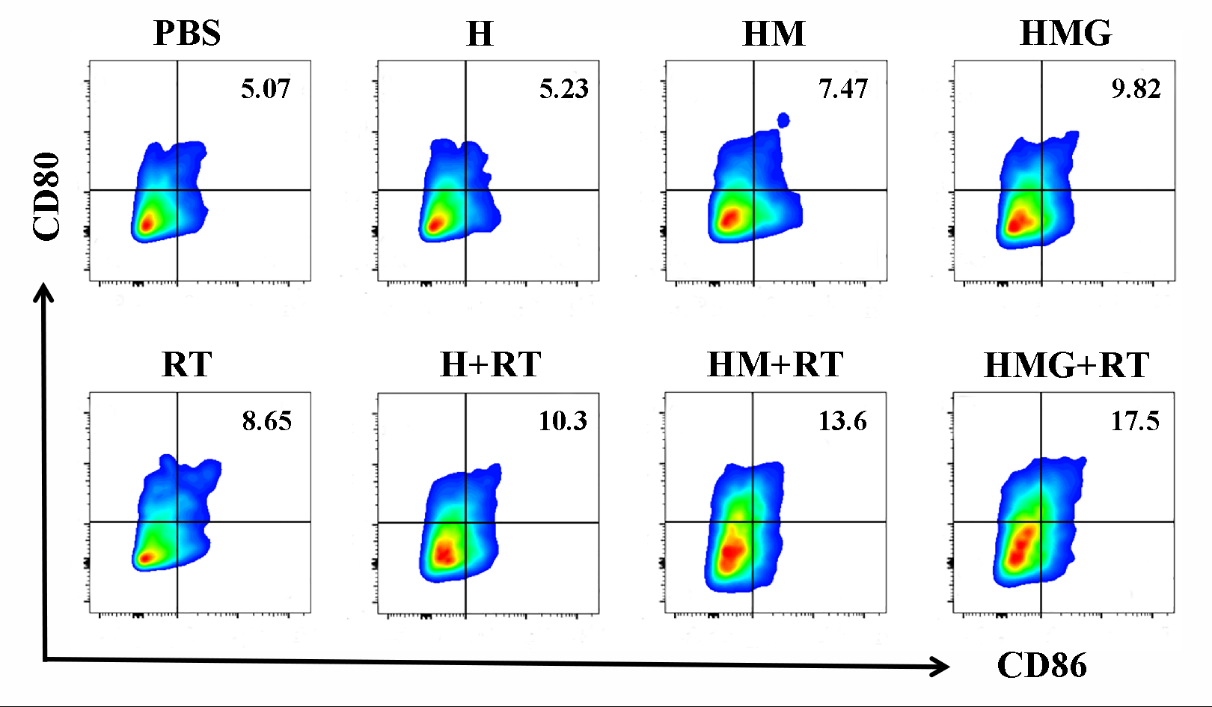


**Fig. S11.** Flow cytometry data of DC maturation (CD80^+^ CD86^+^) from treated tumors.


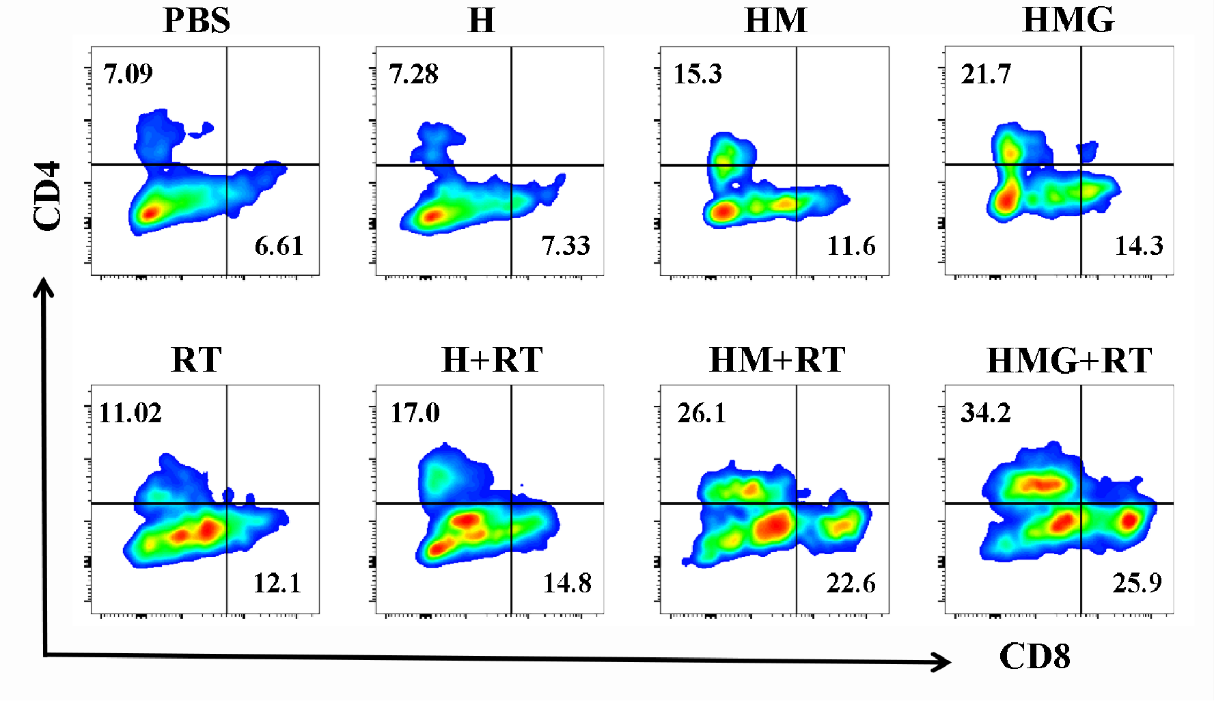


**Fig. S12.** Flow cytometry analysis of intratumor infiltration of CD4^+^ and CD8^+^ T cells from treated tumors.


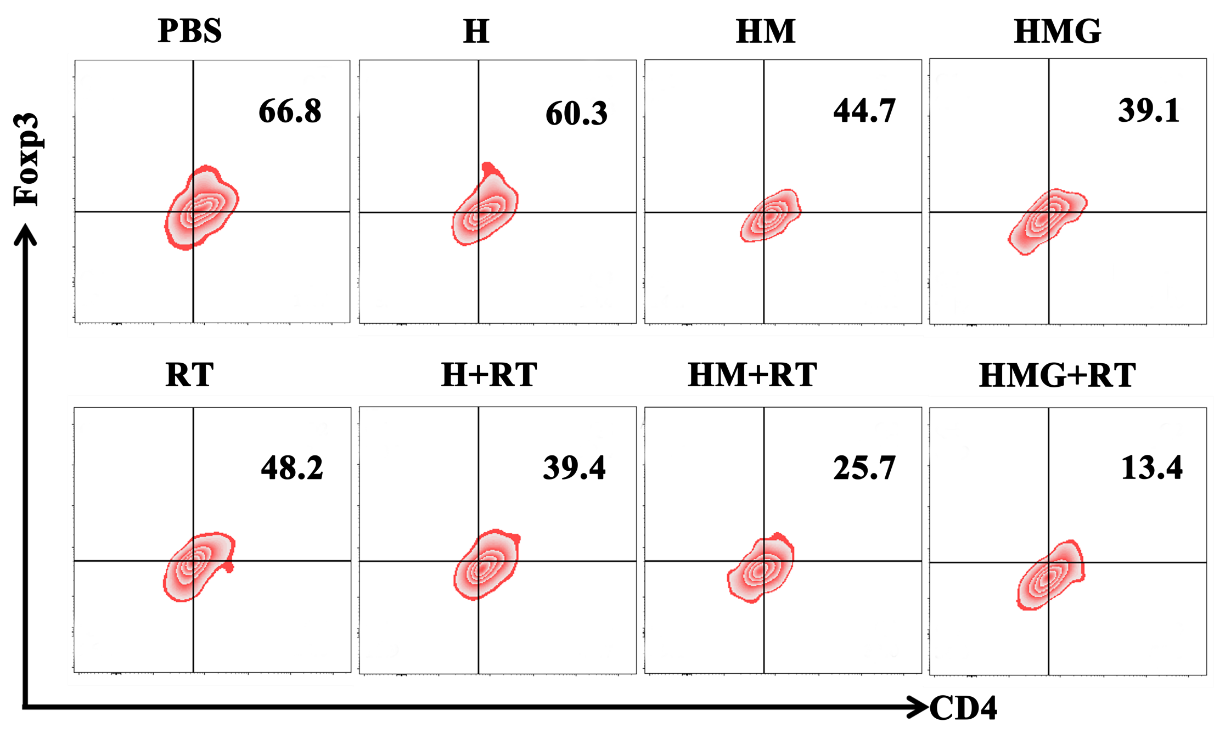


**Fig. S13.** Flow cytometry analysis of CD4^+^ FOXP3^+^ (Treg) from treated tumors.
